# New ^19^F NMR methodology reveals structures of molecules in complex mixtures of fluorinated compounds†

**DOI:** 10.1039/d1sc06057k

**Published:** 2022-02-25

**Authors:** Alan J. R. Smith, Richard York, Dušan Uhrín, Nicholle G. A. Bell

**Affiliations:** EaStCHEM School of Chemistry, University of Edinburgh David Brewster Rd Edinburgh EH9 3FJ UK Nicholle.Bell@ed.ac.uk

## Abstract

Although the number of natural fluorinated compounds is very small, fluorinated pharmaceuticals and agrochemicals are numerous. ^19^F NMR spectroscopy has a great potential for the structure elucidation of fluorinated organic molecules, starting with their production by chemical or chemoenzymatic reactions, through monitoring their structural integrity, to their biotic and abiotic transformation and ultimate degradation in the environment. Additionally, choosing to incorporate ^19^F into any organic molecule opens a convenient route to study reaction mechanisms and kinetics. Addressing limitations of the existing ^19^F NMR techniques, we have developed methodology that uses ^19^F as a powerful spectroscopic spy to study mixtures of fluorinated molecules. The proposed ^19^F-centred NMR analysis utilises the substantial resolution and sensitivity of ^19^F to obtain a large number of NMR parameters, which enable structure determination of fluorinated compounds without the need for their separation or the use of standards. Here we illustrate the ^19^F-centred structure determination process and demonstrate its power by successfully elucidating the structures of chloramination disinfectant by-products of a single mono-fluorinated phenolic compound, which would have been impossible otherwise. This novel NMR approach for the structure elucidation of molecules in complex mixtures represents a major contribution towards the analysis of chemical and biological processes involving fluorinated compounds.

## Abstract 2

^19^F-centred NMR structure determination protocol alleviates the need for compound separation. Disinfection byproducts of chloramination were unraveled by analyzing the reaction pathways of a single fluorinated molecule.

## Graphical abstract

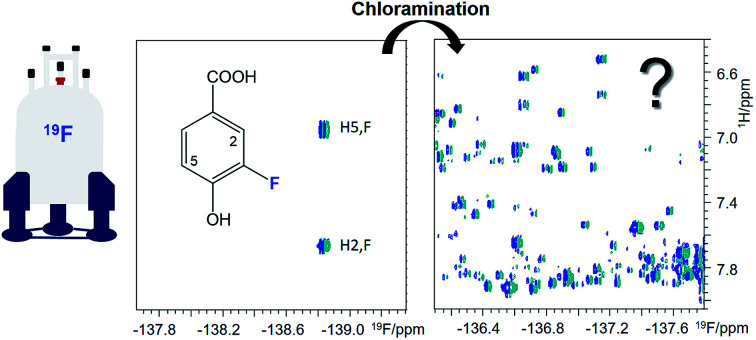

## Introduction

While fluorine-containing compounds are the least abundant natural organohalides,^1^ modern society has become dependent on numerous man-made fluorinated organic molecules such as pharmaceuticals and agrochemicals.

Presently, about 20% of the commercial pharmaceuticals contain fluorine and the proportion of newly approved fluoro-pharmaceuticals is rising steadily.^2–4^ Similarly, fluoro-agrochemicals have become indispensable for crop production and protecting public health from parasitically transmitted infectious diseases;^5^ 53% of all active agrochemicals registered during 1998–2020 are classed as fluoro-agrochemicals.^6^ New fragrance and semiochemical molecules can also benefit from fluorination.^7^ In addition, ^18^F is the most frequently used radioisotope in positron emission tomography radiopharmaceuticals for both clinical and preclinical research, and the search for simple and efficient ^18^F-labeling procedures is an active research area.^8^

Reflecting such interest in fluorinated molecules, design of efficient and environmentally safe fluorination methods^9–11^ and scaled up manufacture of fluorinated molecules^12^ are among the most active fields of organic chemistry. Enzymatic^13^ and chemoenzymatic^14–16^ platforms for the preparation of fluorinated compounds are also emerging. To support these developments, there is a need to characterise fluorinated molecules using efficient analytical methods, amongst which ^19^F NMR spectroscopy plays a prominent role. What makes ^19^F the ideal NMR nucleus is its high sensitivity, 100% natural abundance, large chemical shift dispersion and strong and far-reaching spin–spin interactions.

An important advantage of ^19^F over other nuclei is the absence of the background signal, reflecting the lack of fluorinated endogenous compounds. ^19^F NMR has the ability to study fluorinated molecules in the presence of other CHN-containing molecules and mixtures of fluorinated compounds produced by chemical or chemoenzymatic reactions could in principle be analysed with minimal clean-up steps or compound separation.

In its simplest form, 1D ^19^F NMR has been widely used in studies of biodegradation and biotransformation of fluorinated compounds^17–19^ and has helped to characterise their catabolic pathways^20–24^ and identify cryptic liabilities and features with potentially problematic structural arrangements,^25^ which can lead to recalcitrance and/or toxicity.^26^ Nevertheless, studying biodegradation pathways still typically requires isolation of metabolites and their identification using known standards;^17^ both of these steps could be problematic. Another frequent application of ^19^F NMR comes from using a fluorinated molecule as one of the reactants in studies of mechanisms and kinetics of chemical reactions.^27,28^

The methodology presented here aims to make the process of structure elucidation of fluorine-containing molecules contained in (complex) mixtures more efficient. It follows the “NMR spies” approach, where ^13^C labelled tags provide information about the nuclei in their vicinity,^29,30^ leading to structural characterisation of molecules. In a recent example, introduction of -O^13^CH_3_ groups to a subset of molecules as NMR tags led to structural characterisation of 32 phenolic molecules, or their fragments, in a complex matrix of peat fulvic acid.^31^

In the case of fluorinated organic compounds, ^19^F atoms provide a 100% NMR active tags already present in molecules, enabling ^19^F-centred NMR structure determination. An example of this approach includes the FESTA family of NMR experiments^32–34^ that provide ^1^H–^19^F chemical shift correlation and ^1^H–^19^F coupling constants. The FESTA experiments require selective manipulation of individual ^1^H and ^19^F resonances, which is neither achievable (in particular for ^1^H resonances) nor practical for very complex mixtures, such as investigated here.

We have designed a set of nonselective 2D NMR experiments that use far reaching ^1^H–^19^F and ^19^F–^13^C couplings to obtain ^1^H and ^13^C chemical shifts of nuclei multiple bonds away from the fluorine atom. The same experiments also yield accurate values of ^1^H–^19^F, ^19^F–^13^C and ^1^H–^1^H coupling constants and ^13^C-induced ^19^F isotopic shifts. Put together, the obtained information allows elucidation of fluorine-containing molecular moieties and in favourable cases complete structure determination of small fluorinated molecules.

We have chosen to illustrate this approach on a study of disinfection by-products (DBPs) produced during water treatment. DBPs are formed when disinfectants react with naturally dissolved organic matter (DOM), anthropogenic contaminants, bromide, and iodide during the production of potable water. Approximately 600–700 DBPs have been reported in the literature so far,^35^ some of which exhibit severe health effects.^36,37^ Amongst halogenated DBPs, the focus so far has been on the quantification of trihalomethanes (THMs), haloacetic acids (HAAs) and total organic halides (TOXs).^38–41^ As the known compounds constitute less than 50% of TOXs produced by chlorination and less than 20% by chloramination,^38^ new generations of DBPs are being continually identified and classified for high priority toxicity studies.^35,42^ The commonly used alternative disinfectants to chlorine (ozone, chloramines, and chlorine dioxide) produce lower levels of the four regulated THMs and most HAAs as well as TOXs, however, they increase the concentration of some other priority DBPs.^35,38,43^ Chloramination also incorporates nitrogen into DOM molecules^44^ generating N-containing DBPs,^39,45^ which can be even more toxic than those currently regulated.^37,46^ Chloramination was therefore chosen for this study and ^15^N labelled NH_4_Cl was used in all experiments to prepare ^15^N-containing compounds amenable to NMR studies.

Analytical techniques for the structure determination of DBPs play an important role in this process. Traditional methods, such as liquid/liquid extraction, GC, GC/MS, and solid-phase extraction/MS,^47^ often produce only tentative structures that need validation through the use of authentic chemical standards.^35^ Specialised MS^48,49^ and MS/MS^50,51^ techniques are also being used in this field. Ultrahigh resolution Fourier transform ion cyclotron resonance mass spectrometry (FT-ICR-MS) is making contributions to the characterisation of DBPs at the level of molecular formulae, compound class and functional group classification, including identification of compound classes with the highest DBP formation potential.^52–58^ When ion fragmentation is used more definite structural information can be obtained by MS.^49,51,59^

On the other hand, the use of NMR spectroscopy in the structure determination of DBPs is rare and usually requires some form of compound separation.^60–63^ Here we illustrate the power of ^19^F-centred NMR structure elucidation of fluorinated molecules using a complex mixture of DBPs produced by chloramination of a single fluorine-containing molecule.

## Experimental methodology

### Chloramination

A 500 ml sample was prepared with LC-MS grade water and 50 mg L^−1^ of 3-fluoro-4-hydroxybenzoic acid (1). The solution was buffered to pH 7.2 with phosphate buffer. A ^15^N-monochloramine solution was prepared by slow addition of sodium hypochlorite solution to ^15^NH_4_Cl in a chlorine-to-ammonia ratio of 0.8 mol mol^−1^ and added to the sample in a 3 : 5 mass ratio of carbon: disinfectant, as described previously.^64^ All samples were kept in the dark at 20 °C for 5 days before the addition of excess Na_2_S_2_O_3_ to stop the reaction. The reaction mixture was adjusted to pH 2.0 using HCl before being pumped through PPL SPE cartridges (1 g, 6 ml, Agilent) at a flow rate of ∼5 ml min^−1^. Each cartridge was conditioned using methanol followed by acidified Milli-Q water (pH 2). After adsorption of the sample, the column was washed with acidified water in order to minimise the retention of inorganic species. The cartridge was then allowed to dry before being eluted with methanol. The eluent was rotary evaporated to dryness.

### NMR experiments and instrumentation

Six new NMR experiments were designed and used in this work: ① ^19^F-detected variable-time z-filtered 2D ^1^H, ^19^F HETCOR (Fig. S7†); ② ^19^F-detected 2D ^1^H, ^19^F TOCSY-HETCOR (Fig. S8†); ③ ^1^H-detected 2D ^19^F, ^1^H CP-DIPSI3-DIPSI2 (Fig. S9†); ④and ④′ ^19^F-detected 2D ^19^F, ^13^C (^15^N) HMBC optimised for ^*n*^*J*_FC_ and ^1^*J*_FC_ coupling constants, (Fig. S10† and S11†, respectively), and ⑤ ^1^H-detected 2D H^1^C^*n*^F (Fig. S12†). Apart from ② all other pulse sequences make use of a double inversion adiabatic sweep;^65,66^ the pulse sequence ① uses a z-filter to deliver pure phase multiplets;^67^ the pulse sequence ②, inspired by a 3D TOCSY-HSQC experiment,^68^ incorporates the ^1^H chemical shift labelling followed by a spin-lock period before the magnetisation is transferred to ^19^F for detection; pulse sequence ③ is a simple modification of a 3D ^19^F–^1^H heteronuclear TOCSY edited ^1^H–^1^H TOCSY^69^ that removes the ^1^H chemical shift labelling after the ^19^F → ^1^H transfer; the two HMQC based pulse sequences ④ and ④′ use the echo–antiecho quadrature detection as proposed by Bazzo *et al.*^70^ but eliminate the ^19^F chemical shift evolution and yield pure antiphase ^13^C, ^19^F doublets; experiment ⑤ is a purposely designed reduced dimensionality^71–73^ (3,2)D ^19^F-detected HCF correlation experiment with a simplified polarisation transfer pathway relative to the existing ^1^H-detected triple-resonance HCF experiment.^74^ The full analysis of these experiments will be published elsewhere, however, their most relevant aspect for this work, sensitivity, is analysed in the ESI†.

The reaction product mixture (30 mg) was dissolved in CD_3_OH (180 μL) and placed into a 3 mm NMR tube. Spectra involving ^19^F were acquired on a 500 MHz Bruker Avance III HD NMR spectrometer equipped with a 5 mm QCI-F CryoProbe, while the 1D ^1^H and a 2D ^1^H, ^15^N HSQC spectra were obtained on a 800 MHz AVANCE III NMR spectrometer equipped with a 5 mm TCI cryoprobe. All experiments were performed at 300 K using parameters summarised in Table S1†.

## Results and discussion

### Hardware requirements and design of ^19^F-centered experiments

Historically, pulsing on ^1^H and ^19^F in one NMR experiment, a requirement for all experiments discussed here, was only possible on a limited number of spectrometers.^75^ However, this capability is much more common today. When ^13^C information is sought, three channel NMR spectrometers are required for all but perfluorinated molecules. To boost the sensitivity of such experiments, highly sensitive triple- or quadruple resonance cryoprobes capable of pulsing simultaneously on ^1^H, ^13^C and ^19^F are typically required. Such systems have become more widely available, mainly due to their use in binding studies of biomacromolecules with fluorinated ligands.

The chemical shift correlation experiments involving ^19^F have evolved together with general improvements of liquid-state NMR methodology;^75^ most notably the use of adiabatic ^19^F inversion pulses is now widespread.^66,76–78^ Nevertheless, even some more recent ^19^F experiments yield magnitude mode spectra,^76,78^ provide correlation but not the values of coupling constants,^76^ or contain refocusing periods that generally decrease their sensitivity.^77,78^ Some phase sensitive experiments yield complicated cross peak structures, thereby lowering their sensitivity.^79–81^

The new NMR experiments presented here build on these advances, are phase sensitive and produce cross peaks with a simple pattern that allow identification of active coupling constants. They incorporate adiabatic inversion pulses covering a 100 KHz frequency range, ensuring their optimal performance across a range of ^19^F chemical shifts. The use of a single polarisation transfer interval optimised for ^*n*^*J*_HF_ or ^*n*^*J*_FC_ coupling constants and the elimination of the effects of passive coupling whenever possible, means that they provide chemical shift correlations mediated by a broad range of coupling constants (4–12 Hz ^*n*^*J*_HF_ and 3–26 Hz for ^*n*^*J*_FC_, see Tables S2† and S3). When applicable, they also use ^1^H or ^19^F decoupling in the directly detected periods to simplify cross peaks and to boost the sensitivity.

### Hundreds of DBPs formed by chloramination of a single molecule

**Fig. 1:**
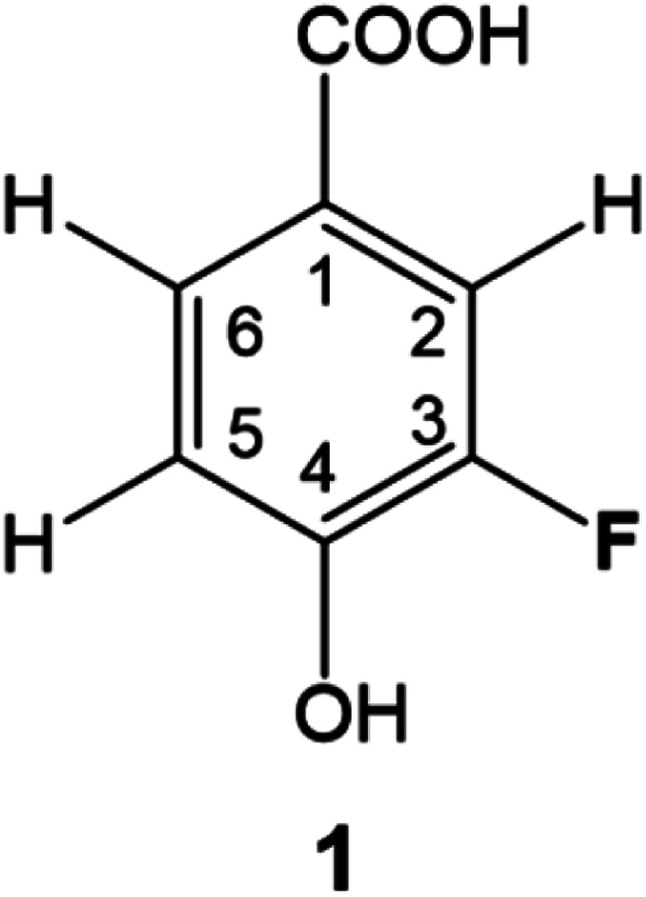
3-Fluoro-4-hydroxybenzoic acid, 1.

DBPs are typically formed from compounds with activated aromatic rings that react with oxidants to produce modified phenolics and unsaturated aliphatic compounds leading to the generation of trihalomethanes.^82^ A simple molecule, 3-fluoro-4- hydroxybenzoic acid (1, Fig. 1) was therefore selected as a suitable model compound for chloramination using ^15^NH_4_Cl.

**Fig. 2:**
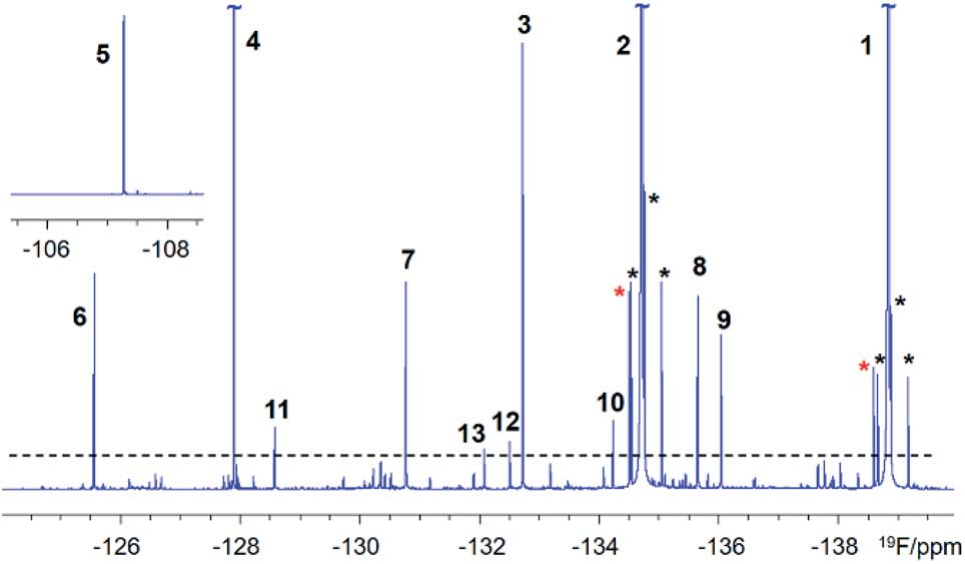
A partial 500 MHz ^1^H-decoupled 1D ^19^F spectrum of the chloramination products of 1. Signals above the dashed line are numbered. Black and red asterisks around the two most intense signals, of 1 (the starting material) and 2 (the major product), indicate ^13^C satellites and their methyl esters as purification by-products, respectively.

A 500 MHz ^1^H-decoupled 1D ^19^F spectrum of the reaction mixture produced by chloramination of 1 is very complex; it contains hundreds of peaks of varying intensity spread across a 90 ppm ^19^F chemical shift range, with the majority and the most intense signals appearing within a 34 ppm range. A partial spectrum is shown in Fig. 2 with thirteen of the most intense resonances numbered. Fig. S1† and S2 present vertical expansions of the full ^19^F spectrum and the aromatic part of a ^1^H NMR spectrum of the reaction mixture, respectively.

Providing fluorine is not removed during the reaction, chloramination products of a fluorinated compound will contain at least one ^19^F atom. If the reaction causes oligomerisation, molecules with several ^19^F atoms will also be present. Nevertheless, these will likely be too distant to exhibit ^19^F–^19^F couplings and ^19^F atoms will therefore only couple to protons in ^12^C molecules and protons and carbons in ^13^C isotopomers. In molecules that incorporated ^15^N, couplings of ^19^F with ^15^N could arise. The ^19^F atom thus represents a convenient ‘spy’ that reports on the ^19^F, ^1^H, ^13^C and ^15^N NMR chemical shifts and numerous coupling constants of fluorinated molecules, underpinning the structural characterisation of DBPs.

### ^19^F, ^1^H and ^13^C chemical shifts and *J* couplings determination

**Fig. 3:**
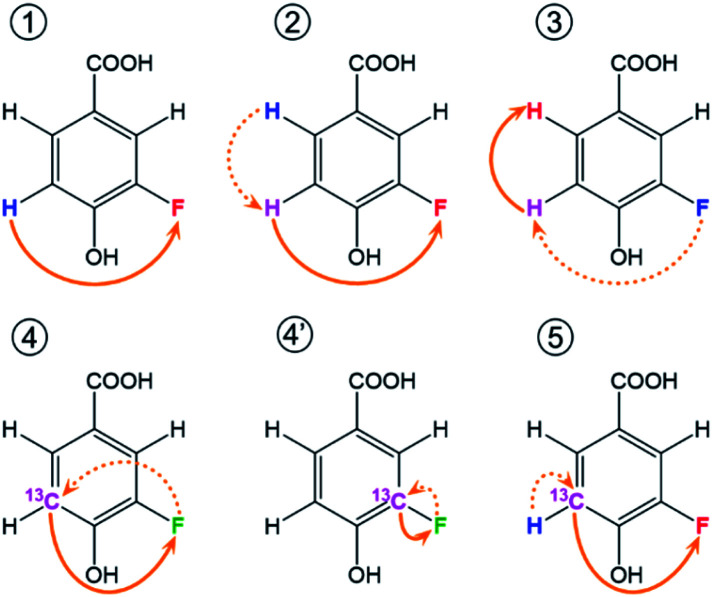
Schematic representation of ^19^F-centred NMR experiments. The blue, pink and red colours represent the starting, intermediary and the detected nucleus for one example of the magnetization transfer pathways, while green is used when both the starting and detected nucleus are the same. These pathways are used by the following experiments: ① ^19^F-detected z-filtered 2D ^1^H, ^19^F HETCOR; ② 2D ^1^H, ^19^F TOCSY-HETCOR; ③ 2D ^19^F, ^1^H CP-DIPSI3-DIPSI2; ④ 2D ^19^F, ^13^C (^15^N) HMBC optimised for ^*n*^*J*_FC_ (^*n*^*J*_FN_) coupling constants; ④′ 2D ^19^F, ^13^C HMBC optimised for ^1^*J*_FC_ coupling constants; ⑤ (3,2)D H^1^C^*n*^F correlation experiment. Dashed and full orange arrows connect the initial and final magnetisation transfer steps, respectively.

Extensive spin–spin interactions involving ^19^F open numerous magnetisation transfer pathways (Fig. 3) that can be exploited to yield chemical shift correlations of many nuclei.

**Fig. 4:**
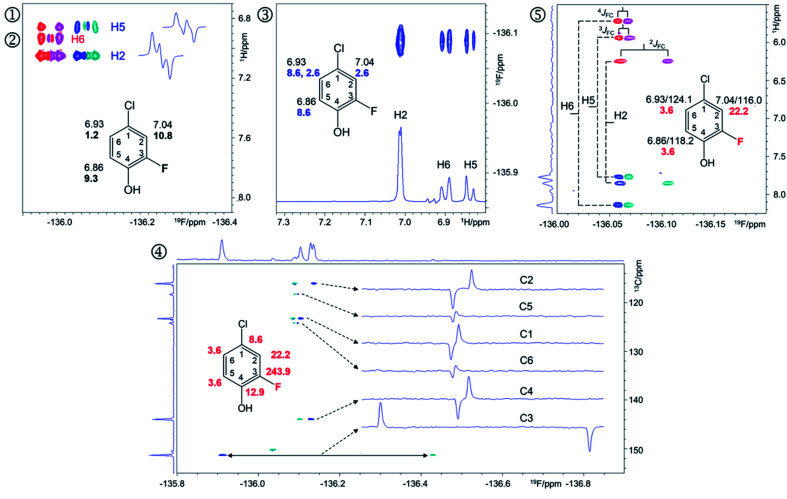
Regions of the 500 MHz NMR spectra acquired with the pulse sequences presented in Fig. S8–S12† showing chemical shift correlations for compound 9. In addition to 2D cross peaks, the figures display the structure of 9 with selected NMR parameters, and where appropriate, *F*_2_ traces showing the fine structure of cross peaks. ① Overlay of the 2D ^1^H, ^19^F HETCOR (blue/turquoise) and ② 2D ^1^H, ^19^F TOCSY-HETCOR (red/magenta) cross peaks. The TOCSY spectrum was left-shifted to facilitate identification of signals. *F*_2_ traces through H2 and H5 cross peaks from the HETCOR spectrum are shown. ^1^H chemical shifts and *J*_HF_ values (bold) are displayed on the structure; ③ A 2D ^19^F, ^1^H CP-DIPSI3-DIPSI2 spectrum; *F*_2_ trace at the ^19^F chemical shift of 9 is shown; ^1^H chemical shifts and *J*_HH_ values (blue) are displayed on the structure; ④ A 2D ^19^F, ^13^C HMBC spectrum optimised for ^*n*^*J*_FC_ coupling constants. Internal *F*_1_ and *F*_2_ projections and *F*_2_ traces at the ^13^C chemical shifts of 9 are displayed; the *J*_FC_ values are shown in red; ⑤overlay of two edited 2D(3,2) H^1^C^*n*^F correlation spectra containing individual cross peaks of the *F*_1_ doublets that code for ^13^C chemical shifts. Blue/turquoise and red/magenta colours indicate antiphase *J*_FC_*F*_2_ doublets in each spectrum. The internal *F*_1_ projection of one of the spectra is displayed. Vertical lines connect the corresponding signals with their midpoint marking the ^1^H chemical shifts. The ^1^H/^13^C chemical shifts and *J*_FC_ coupling constants (red) are indicated. These active coupling constants appear in antiphase, which can cause partial signal cancellation. Thus, to obtain more accurate values it is best to determine them from a ^1^H coupled ^19^F spectrum. The H6,F cross peak only appears in the 2D ^1^H, ^19^F TOCSY-HETCOR spectrum (②, Fig. 4) because the *J*_H6,F_ coupling constant is too small to generate a response in the former experiment. A 2D ^19^F, ^1^H CP-DIPSI3-DIPSI2 (③, Fig. 4) serves to extend the proton networks beyond the protons coupled to ^19^F, similarly to 2D ^1^H, ^19^F TOCSY-HETCOR experiment. However, as a ^1^H-detected experiment, it provides values of *J*_HH_ coupling constants that are beneficial to the structure determination process. A 2D ^19^F, ^13^C HMBC spectrum optimised for ^*n*^*J*_FC_ coupling constants (④, Fig. 4) provides the chemical shifts and ^n^*J*_FC_ coupling constants of all ^19^F-coupled carbons. For one-bond ^19^F–^13^C correlations, the sensitivity of the experiment can be enhanced by optimising the polarisation transfer periods for ^1^*J*_FC_ coupling constants (pulse sequence of Fig. S11†). If the values of ^1^*J*_FC_ coupling are known, the HMBC experiment can be set up to yield the one-bond correlations as well. Finally, the outcome of a simultaneous H^1^C^*n*^F correlation is illustrated in ⑤ (Fig. 4). This intrinsically 3D experiment has been modified using the principles of reduced dimensionality^83,84^ to produce a (3, 2)D experiment. Here, the ^13^C chemical shift is coded in the ^1^H dimension by the width of the *F*_1_-doublet. In this experiment two interleaved spectra are acquired, which contain in-phase or antiphase *F*_1_ doublets. Editing of these spectra increases the S/N ratio and removes half of the cross peaks in each spectrum, thus reducing spectral overlap.

A 2D ^1^H, ^19^F correlation spectrum (Fig. S3†) illustrates the complexity of the investigated mixture. Zoomed in regions of ^19^F-centred spectra acquired in this work showing the assignment of signals of compound 9 are presented in Fig. 4.

The ^19^F-detected z-filtered 2D ^1^H, ^19^F HETCOR spectrum (①, Fig. 4) shows HF cross peaks with protons H2 and H5 whose appearance is mediated by large *J*_HF_ coupling constants.

### Sensitivity and resolution limits of ^19^F-centered NMR

Based on the analysis of signal intensities of the thirteen most intense resonances seen in the ^1^H-decoupled 1D ^19^F NMR spectrum of a 30 mg mixture (Fig. 1 and S1†), it can be estimated that compound 11 – the lowest concentration compound that yielded signals in experiments involving ^13^C – is present at 1 mM (or 30 μg in 180 μL of CD_3_OH in a 3 mm NMR tube assuming an average molecular weight of 170 g mol^−1^ for compounds in this mixture). This sensitivity limit applies to an overnight experiment on a 500 MHz NMR spectrometer equipped with a 5 mm QCI-F CryoProbe and a 3 mm sample tube.

Exploring a hypothetical scenario, 30 mg of a mixture could contain a 1000 similar size compounds at around 30 μg each. These would be amenable to the structure determination as outlined here, thanks to the remarkable sensitivity of today's NMR spectrometers and the efficiency of the ^19^F-centered approach. The sensitivity of ^1^H, ^19^F correlation experiments is naturally higher with an estimated concentration limit of ∼30 μM (or 1 μg for compounds with *M*_w_ = 170 g mol^−1^ in 180 μl). This statement is supported by the appearance of hundreds of cross peaks in the 2D ^1^H, ^19^F HETCOR spectrum (Fig. S3†) associated with ^19^F signals that are 30 × weaker than the signal of 11. Around 200 spin systems of these minor compounds could be identified in this spectrum. Their cross peaks were resolved due to the exquisite sensitivity of ^19^F to its chemical environment. The presented analysis thus provides a glimpse into the complexity of mixtures that are amenable to structure elucidation by ^19^F-centered NMR.

### Structure determination process in ^19^F-centred NMR

**Fig. 5:**
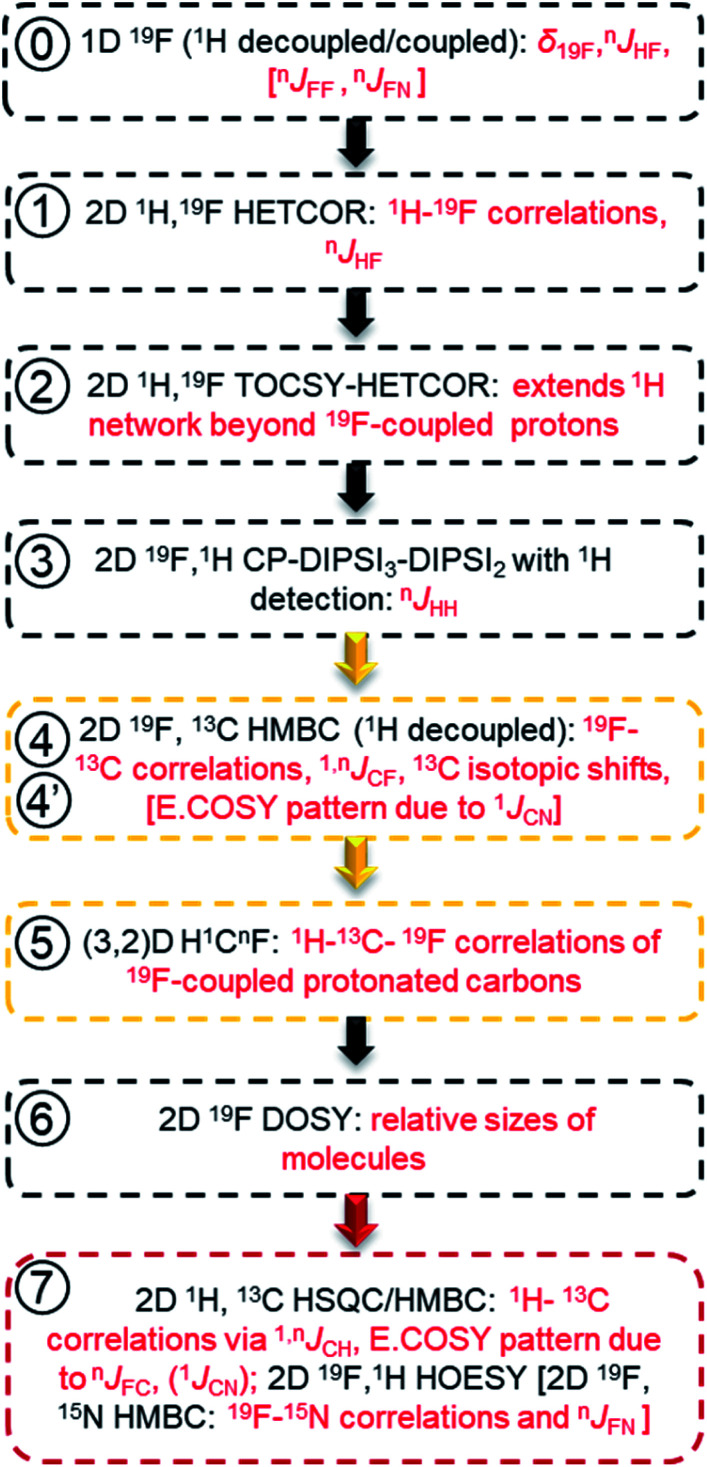
Flow chart for acquiring and working with spectra of mixtures of fluorinated compounds. The information obtained is given in red. Golden and red boxes denote experiments involving ^13^C, and the experiments for extending the structure beyond the F-containing moieties, respectively.


In reference (and using symbols 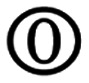 to ⑦) to the schematic representation of ^19^F-centred NMR experiments (Fig. 3) and the example spectra of the chloramination product mixture (Fig. 4), the steps involved in ^19^F-centred NMR structure determination are discussed below and summarised in a flowchart (Fig. 5).

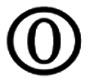
 The process starts with the acquisition of standard 1D ^1^H-coupled and ^1^H-decoupled ^19^F spectra, which provide ^19^F chemical shifts and values of ^*n*^*J*_HF_ coupling constants.

① Chemical shifts of ^19^F-coupled protons are determined in a 2D ^19^F, ^1^H HETCOR experiment; ^n^*J*_HF_ coupling constants are assigned.

② The ^19^F-associated proton network is extended by protons not directly coupled to ^19^F in a 2D ^19^F, ^1^H TOCSY-HETCOR experiment.

③ *J*_HH_ coupling constants are obtained in a 2D ^19^F, ^1^H CP-DIPSI3-DIPSI2 experiment; extension of the proton network, established by ①and ②, is possible.

The correlated ^19^F and ^1^H chemical shifts and homo- and heteronuclear coupling constants can now be interpreted to propose structural fragments by considering the effect of substituents,^85^ values of *J*_HF_ coupling constants^86,87^ (Table S2†) and *J*_HH_ coupling constants.

④ 2D ^19^F, ^13^C HMBC experiment provides ^19^F–^13^C chemical shift correlations, values of ^1,*n*^*J*_CF_ coupling constants and ^13^C-induced ^19^F isotopic shifts.

⑤ The 2D(3,2) H^1^C^n^F correlation spectra provide a distinction between protonated and non-protonated ^19^F-coupled carbons and chemical shift correlations of HC pairs.

Experiments involving ^19^F–^13^C correlations are very informative and should be performed if sufficient amount of material is available. Considering the effects of substituents,^88^ the sizes of *J*_FC_ coupling constants^86,87^ (Table S3†) and ^13^C-induced ^19^F isotopic chemical shifts (Table S4†), structural fragments proposed by the analysis of ^1^H/^19^F data can be verified and extended.

⑥ Relative sizes of molecules in a mixture are estimated by a 2D ^19^F DOSY experiment.

Taking advantage of the large chemical shift dispersion of ^19^F, interpretation of ^19^F-detected DOSY spectra^89^ (Fig. S4†) is straightforward due to minimal signal overlap. A one-shot DOSY experiment^90^ with rectangular ^19^F pulses was used here; for spectra covering a wider range of ^19^F chemical sifts, the use of adiabatic pulses is recommended.^91,92^ For the studied mixture, the measured diffusion coefficients generally decreased with increasing molecular weight of compounds and their substituents in the order COOH, NO_2_ and Cl. The contribution from the carboxyl groups was particularly large, presumably because of the formation of hydrogen bonds with the solvent. Assessment of the molecular weight also helps to decide if data beyond the reach of ^19^F-centred experiments are required.

⑦ 2D ^1^H, ^13^C HSQC/HMBC spectra provide one-bond and long-range ^1^H–^13^C correlations beyond the reach of the ^19^F-centered experiment. 2D ^19^F, ^1^H HOESY experiments can also help to identify more remote protons.

Using standard 2D ^1^H, ^13^C one-bond and long-range correlated experiments alone to analyse complex mixtures is problematic due to the complexity of their spectra. Nevertheless, for larger molecules, which contain spin systems isolated from those containing ^19^F, protons and carbons identified by ^19^F-centred experiments can act as starting points for extending the assignments through the analyses of 2D ^1^H, ^13^C HSQC/HMBC spectra. Similarly, 2D ^19^F, ^1^H HOESY experiments^75,93^ can reach more remote protons by utilising ^19^F, ^1^H NOEs.

Due to use of ^15^NH_4_Cl, some fluorinated compounds studied here, contain ^15^N, which opened another route for obtaining structural information as summarised using square brackets in the flow chart of Fig. 5. The ^19^F–^15^N chemical shift correlations can be obtained by a 2D ^19^F, ^15^N HMBC experiment (Fig. S10 and S5†). For nitrogen-containing DBPs, carbons directly bonded to ^15^N are identifiable by the E.COSY pattern of cross peaks in 2D ^19^F, ^13^C HMBC spectra caused by relatively large ^1^*J*_NC_ (11–13 Hz) coupling constants. The sizes of *J*_FN_ (or *J*_FF_) coupling constants are best determined from 1D ^1^H-decoupled ^19^F spectra. A potential presence of ^19^F–^19^F interactions can be probed by a 2D ^19^F, ^19^F COSY experiment.

### Analysis of the chloramination reaction pathways

**Fig. 6:**
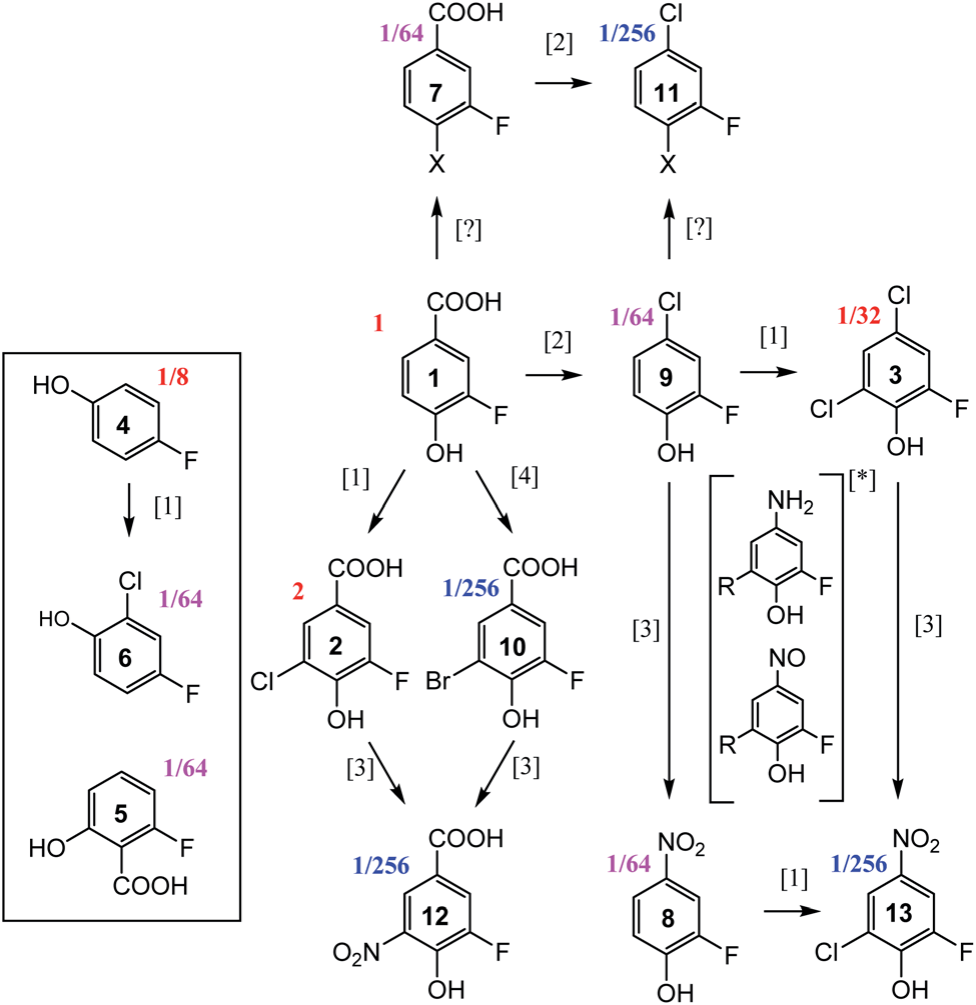
Reaction pathways [1],^94^ [2],^96^ [3],^50^ and [4],^95^ identified in chloramination of 1. Compounds enclosed in a rectangle fall outside of this classification. Fractions given represent concentrations relative to the starting material, 1, as estimated from the intensity of signals in the 1D ^19^F NMR spectrum. *unconfirmed intermediates, R = H or Cl.

^19^F-centred NMR methodology provided a rich set of NMR parameters for the chloramination reaction product mixture (Table S5†), which allowed the structure elucidation of eleven molecules, present in concentrations above the current sensitivity threshold, and partial structures for two additional molecules (Fig. 6). The analysed mixture was prepared in a 5 day experiment, which led to extensive modification of the starting material producing phenolic and likely also non-phenolic compounds, initially *via* transfer of Cl released from hypochlorous acid, HOCl.^50,56^ Electrophilic substitution reactions, as the main chlorination mechanism for aromatic substitution,^94^ resulted in chlorination of 1 producing 2 as the major product. Several DBPs generated by other reactions were also modified in this way–a Cl substitution at the activated ortho position next to an OH group (9→3, 8→13, 4→6). The unexpected appearance of a brominated compound formed from the starting material (1→10) can be explained by the use of NaOCl manufactured by the electrolysis of sodium chloride. Water used in this process contains small amounts of sodium bromide,^95^ which led to the production of sodium bromate – the source of Br. The presence and the position of Br in compound 10 was established through a comparison of chemical shifts of 2 and 10, which differ only in the nature of the halogen substituent. The experimental differences in the ^1^H and ^13^C NMR chemical shifts at corresponding positions agreed perfectly with the values predicted by considering the effects of Cl and Br on the chemical shift of benzene resonance.^76,79^ In addition, peaks at *m*/*z* 232.9255 and 234.9235, corresponding to [C_7_H_3_O_3_F^79^Br]^−^ and [C_7_H_3_O_3_F^81^Br]^–^ ions, were detected in FT ICR MS spectra of the product mixture (data not shown), confirming the presence of Br in compound 10.

The second reaction type observed was decarboxylative chlorination^96^ (1→9 or 7→11). The halogenated sites also continued to react with monochloramine through nucleophilic substitution by H_2_N in a dechlorinative amination.^97^ The generated aromatic amines were further oxidised by NH_2_Cl to form nitroso- and eventually nitro compounds,^50^ (2→12, 10→12, 9→8, 3→13). An unexpected outcome was the appearance of compounds 4 and 5. These compounds were not part of the starting material, as confirmed by the absence of their signals in the ^1^H-decoupled ^19^F spectrum of 1. Their structures were verified by a comparison of NMR parameters with literature data.^98,99^ Performing such checks is generally recommended, especially in instances where the appearance of the identified compounds is difficult to rationalise. Such comparisons are considered to be reliable due to sensitivity of NMR parameters to molecular structures.

Two additional compounds, containing a tri-substituted benzene ring with a carboxylic group (7) or a chlorine (11) at position C-1, were identified. The differences between the ^13^C and ^1^H chemical shifts of the corresponding atoms of these compounds matched the differences observed for an analogous pair of molecules, 1 and 9. A possible mechanism for the formation of compounds 7 and 11 from 1 and 9, respectively, is *via* resonance stabilised phenoxyl radicals produced by dissociation or abstraction of the phenolic hydrogen.^100^ This hypothesis is supported by the observed changes of colour of the reaction mixture over the course of 5 days, which could indicate the existence of quinone/semiquinone equilibria. Based on the ^19^F DOSY spectrum (Fig. S4†), molecules 7 and 11 are the largest, likely dimeric molecules. Attempts to extend their structures using ^1^H, ^13^C correlation experiments, as suggested in step ⑦ of Fig. 5, did not yield further information. A ^1^H, ^19^F HOESY experiment (not performed here) represents another opportunity for structural characterisation.

The origin of most but not all compounds identified in this study can thus be explained by known reaction mechanisms. It is possible that during the course of chloramination, fluorine radicals were created, further modifying the pool of the produced compounds. This could help to explain the variety of ^19^F containing compounds (Fig. 1 and ES1†) that are present in concentrations too low to currently allow their structure elucidation. The other source of heterogeneity of the final mixture are the N-containing molecules, as indicated by the richness of its 2D ^1^H,^15^N HSQC spectrum (Fig. S6†). None of compounds 2–13 contain a protonated NH_*x*_ (*x* = 1, 2) group, indicating that the nitrogenated products of 1 are present at low concentrations.

The number of compounds obtained in our experiments, which admittedly aimed to maximise the production of DBPs, is astounding. Their structural studies will continue to attract attention due to the potential influence of DBPs on human health and the environment.

## Conclusions

By analysing a complex mixture of DBPs produced by chloramination of a single fluorine-tagged molecule, we have demonstrated the feasibility of ^19^F-centred NMR structure determination of small molecules without the need for compound separation. The ^19^F-centred experiments correlated ^19^F chemical shifts with those of ^1^H, ^13^C and ^15^N, provided values of *J*_HF_, *J*_FC_ and *J*_NF_ coupling constants, including ^1^H–^1^H chemical shift correlations and *J*_HH_ coupling constants for a subset of protons. The proposed experiments, which can also be used in their own right, thus collectively represent an efficient NMR approach to the structure determination of mono-fluorinated moieties and small compounds in complex mixtures.

## Data availability

Data is available on request.

## Author contributions

NGAB proposed the methodology, designed experiments and performed the structure elucidation. AJRS, DU and RY contributed to the implementation of the experiments and acquisition of spectra. AJRS performed the chloramination reaction. All authors contributed to the analysis of the spectra and writing of the manuscript.

## Conflicts of interest

There are no conflicts to declare.

## Supplementary Material

SC-013-D1SC06057K-s001
