# Supplementary material for: New 19F NMR methodology reveals structures of molecules in complex mixtures of fluorinated compounds
Source: Chem Sci. 2022 Feb 25;13(13):3766–74. doi: 10.1039/d1sc06057k (PMC8966635; doi:10.1039/d1sc06057k)
Supplement: SC-013-D1SC06057K-s001 [file SC-013-D1SC06057K-s001.pdf]

This journal is © The Royal Society of Chemistry 2022

Supplementary Information

New ^{19}F NMR methodology reveals structures of molecules in complex mixtures of fluorinated compounds

Alan J. R. Smith, Richard York, Dušan Uhrín and Nicholle G. A. Bell*

EaStCHEM School of Chemistry, University of Edinburgh, David Brewster Rd, Edinburgh, EH9 3FJ, UK.

Corresponding Author: Nicholle Bell - School of Chemistry, University of Edinburgh, Joseph Black Building, Kings Buildings, David Brewster Road, EH93FJ. Email: Nicholle.Bell@ed.ac.uk, Orcid: 0000-0001-7887-2659

Table of Contents

Fig. S1. Vertical expansions of a 500 MHz ^{1}H-decoupled 1D ^{19}F spectrum of the reaction mixture of chloraminated 3-fluoro-4-hydroxybenzoic acid, **1**……………………………….……2

Fig. S2. Aromatic region of a 800 MHz ^{1}H NMR spectrum of the reaction product mixture after chlorination of **1**……………………………………………………………….………3

Fig. S3. Partial 2D ^{1}H, ^{19}F HETCOR spectrum of the reaction product mixture..……..............3

Fig. S4. 2D ^{19}F DOSY spectrum of the reaction product mixture………………………..….......4

Fig. S5. 2D ^{19}F, ^{15}N HMBC spectrum of the reaction product mixture ...…………...………..4

Fig. S6. 800 MHz 2D ^{1}H, ^{15}N HSQC spectrum of the reaction product mixture …...…...........5

^{19}F-centered pulse sequences

Fig. S7. Variable-time z-filtered ^{19}F-detected 2D ^{1}H, ^{19}F HETCOR……………………………..6

Fig. S8. Variable-time ^{19}F-detected 2D ^{1}H, ^{19}F TOCSY-HETCOR……………………………...7

Fig. S9. 2D ^{19}F, ^{1}H CP-DIPSI3-DIPSI2……………………………………………..…….........8

Fig. S10. 2D ^{19}F, ^{13}C HMBC optimised for $^{n}J_{FC}$ coupling constants……………………………9

Fig. S11. 2D ^{19}F, ^{13}C HMBC optimised for $^{1}J_{FC}$ coupling constants…………………………...10

Fig. S12. (3, 2)D H^{1}C^{n}F……………………………………………………………………….11

Tables

Table S1. Parameters of NMR experiments performed on the reaction mixture……….…....12

Table S2. Summary of $^{n}J_{HF}$ coupling constants for the 13 characterised compounds….…....12

Table S3. Summary of $^{1,n}J_{FC}$ coupling constants for the 13 characterised compounds…… 13

Table S3. Summary of ^{13}C-induced chemical shifts for the 13 characterised compounds....13

Table S5. Summary of ^{19}F, ^{1}H, ^{13}C and ^{15}N NMR parameters for the 13 characterised compounds…………………………………………………………………………….14

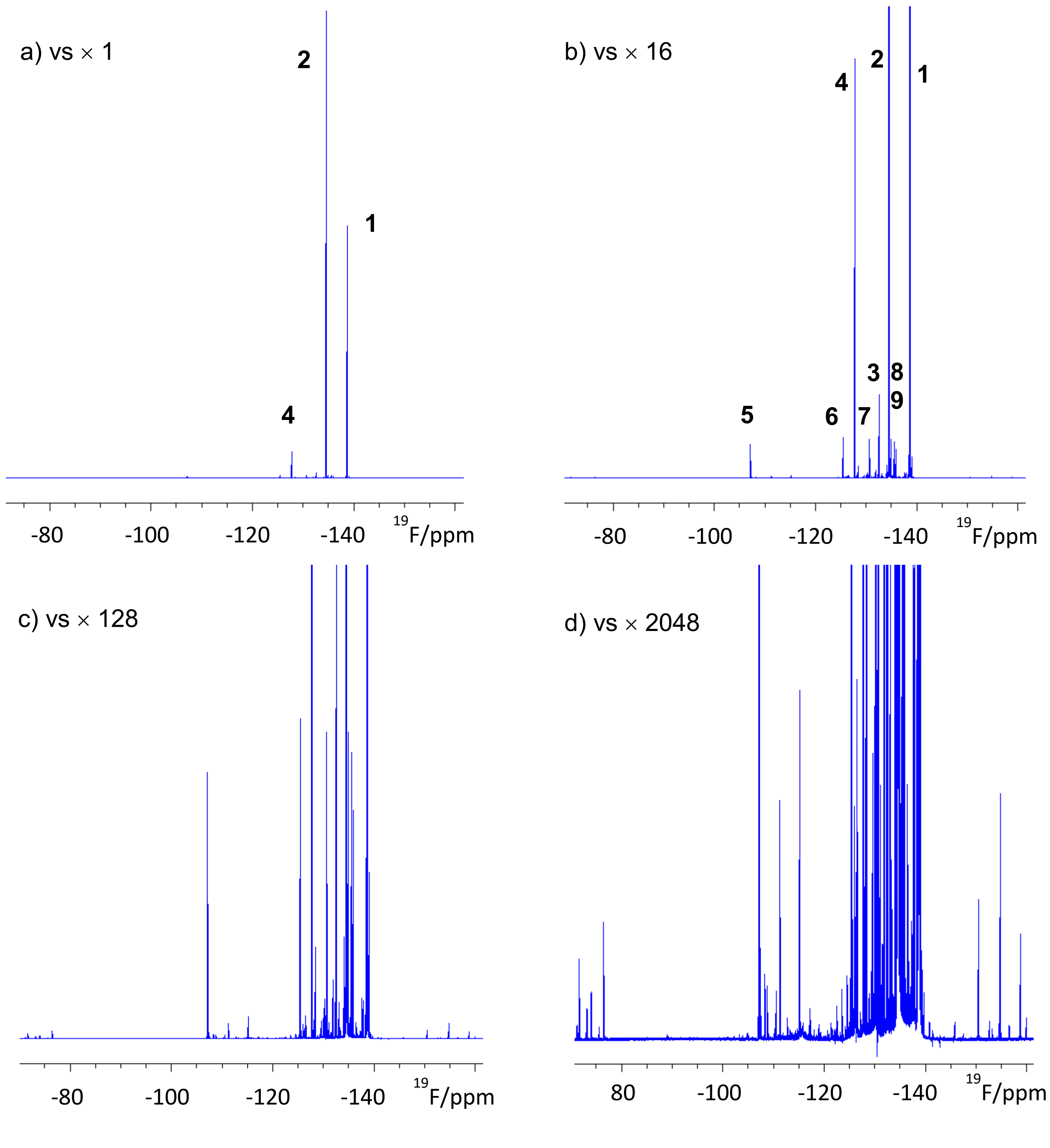

Fig. S1. A 500 MHz ^{1}H-decoupled 1D ^{19}F spectrum of a mixture produced by chloramination of 3-fluoro-4-hydroxybenzoic acid (**1**) using $^{15}NH_4Cl$. (a)-(d) show vertical expansions scaled as indicated.

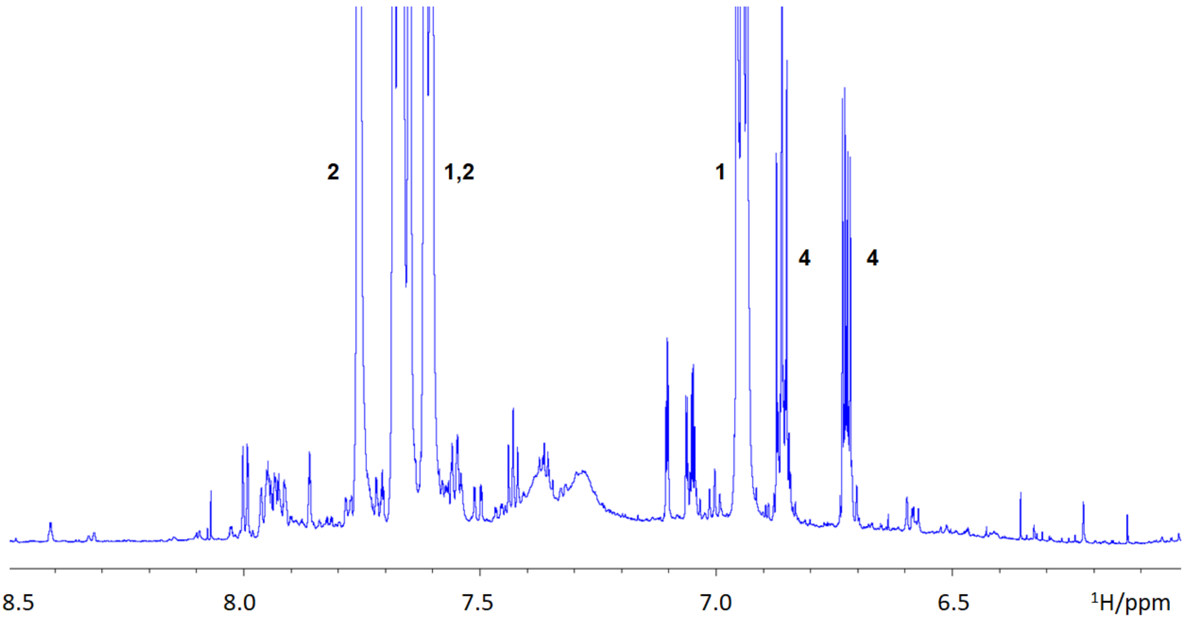

Fig. S2. Aromatic region of an 800 MHz 1D ^{1}H spectrum of a mixture produced by chloramination of 3-fluoro-4-hydroxybenzoic acid (**1**) using $^{15}NH_4Cl$. The spectrum is dominated by the signals of major compounds **1**, **2** and **4**.

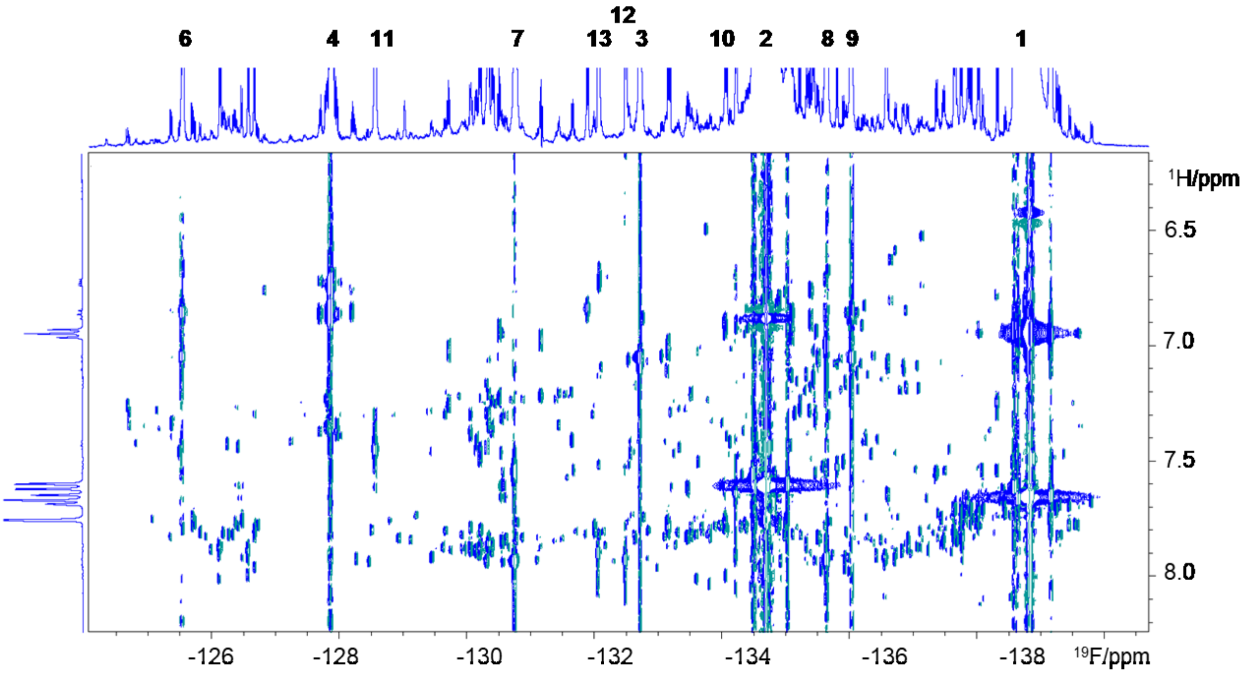

Fig. S3. A large section of a 2D ^{1}H, ^{19}F HETCOR spectrum of the mixture produced by chloramination of **1**.

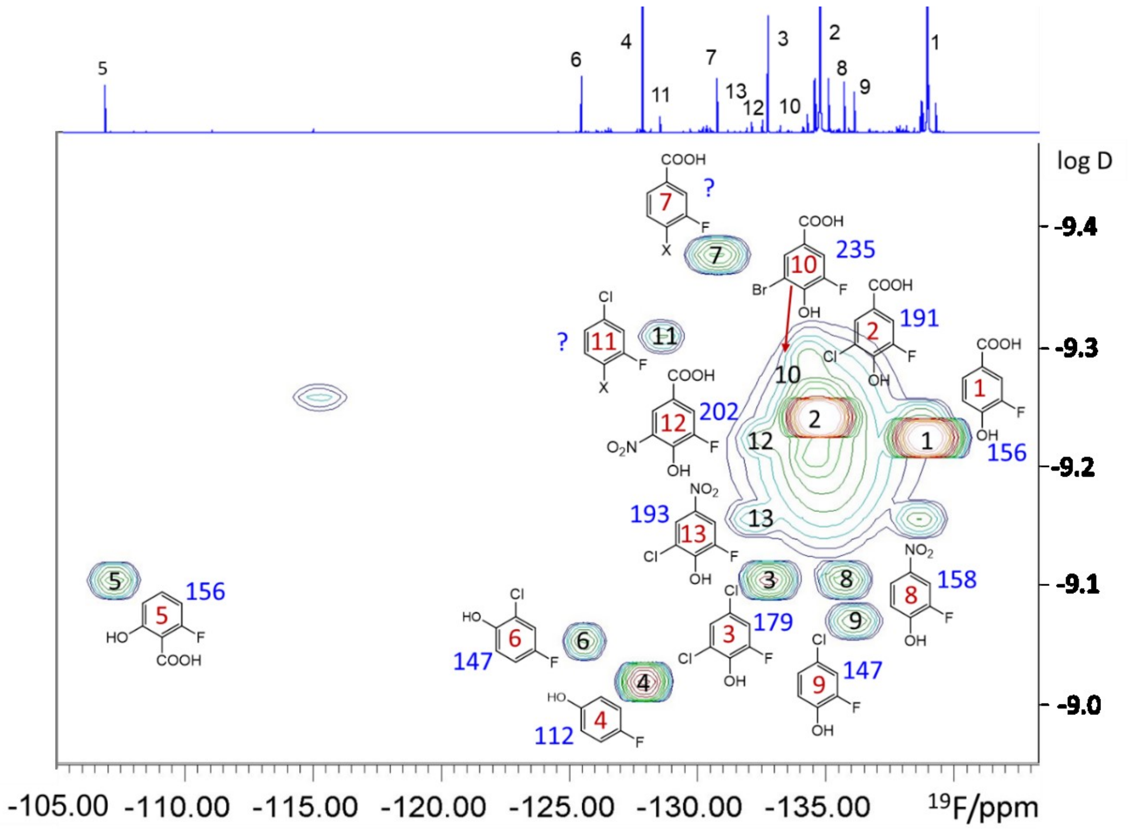

Fig. S4. 2D ^{19}F DOSY spectrum of the reaction product mixture.

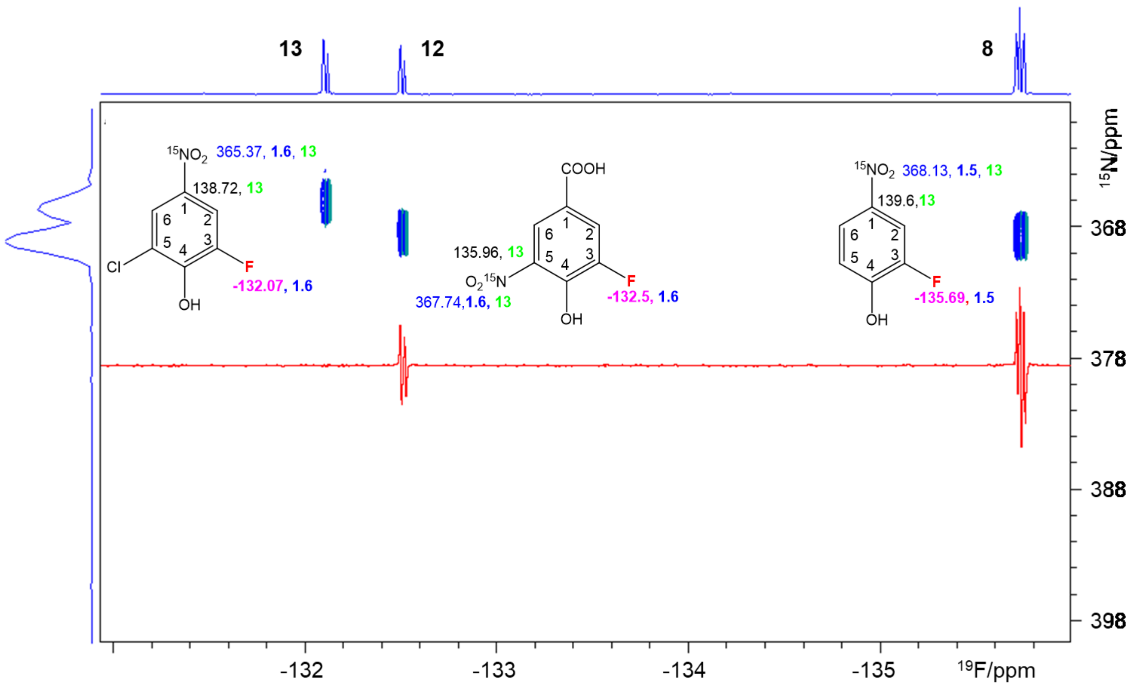

Fig. S5. Partial 2D ^{19}F, ^{15}N HMBC spectrum of the reaction product mixture acquired with the pulse sequence of **Fig. S10** without the ^{1}H decoupling. Internal positive projections are shown on the top and the side of the spectrum respectively. The F_2 trace at 368 ppm in red shows antiphase ($^{n}J_{FN}$) multiplets with inphase splittings ($^{n}J_{HF}$) from **12** and **8**.

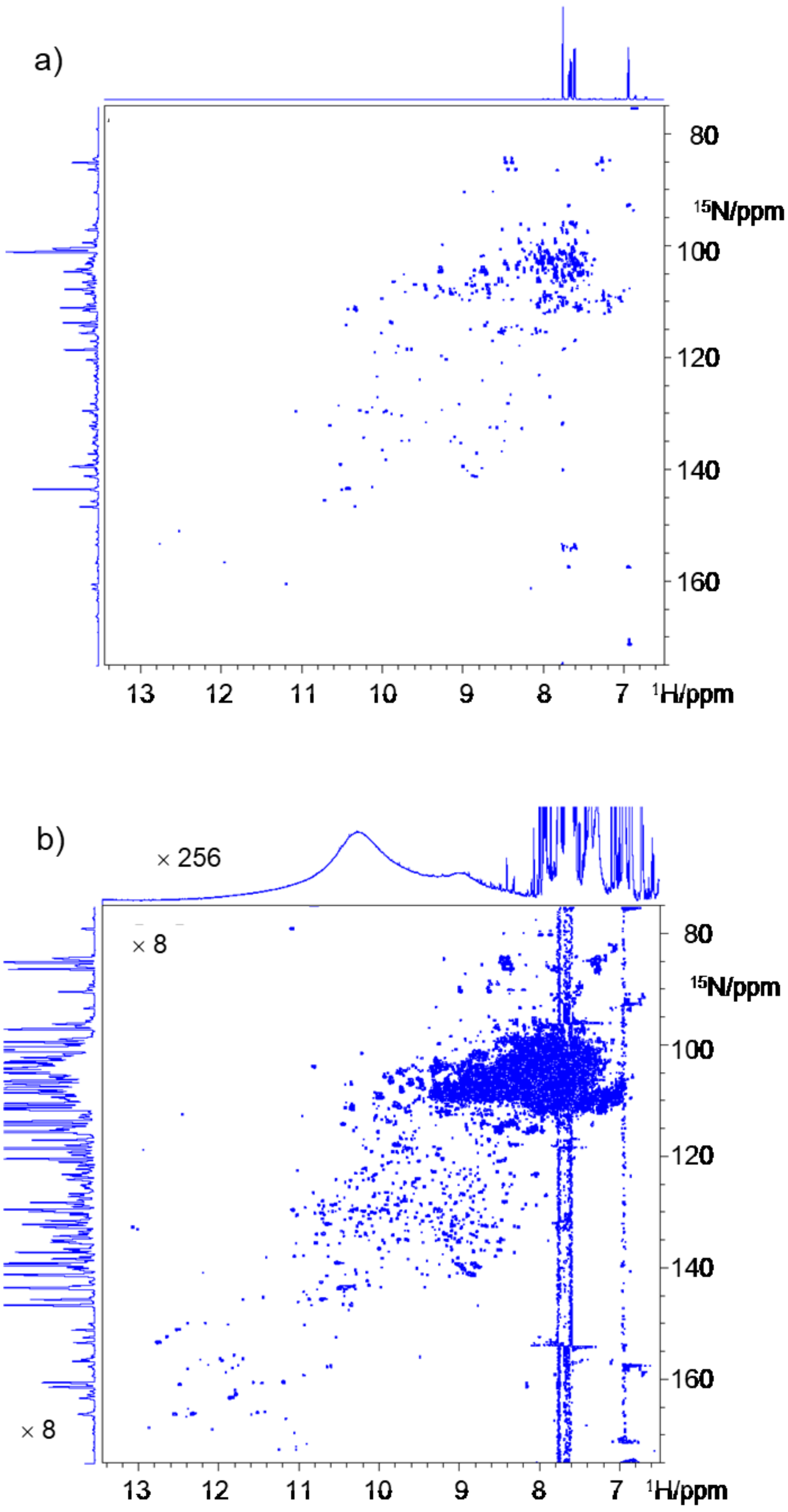

Fig. S6. 800 MHz 2D ^{1}H, ^{15}N HSQC spectrum of the mixture produced by chloramination of **1** using $^{15}NH_4Cl$. (b) represents a vertical expansion of (a) as stated in parts of the spectrum. F_1 noise visible in (b) is due to intense signals of compounds **1** and **2**.

Timing and phase cycling of r.f. pulses of the ^{19}F-centered pulse sequences.

①

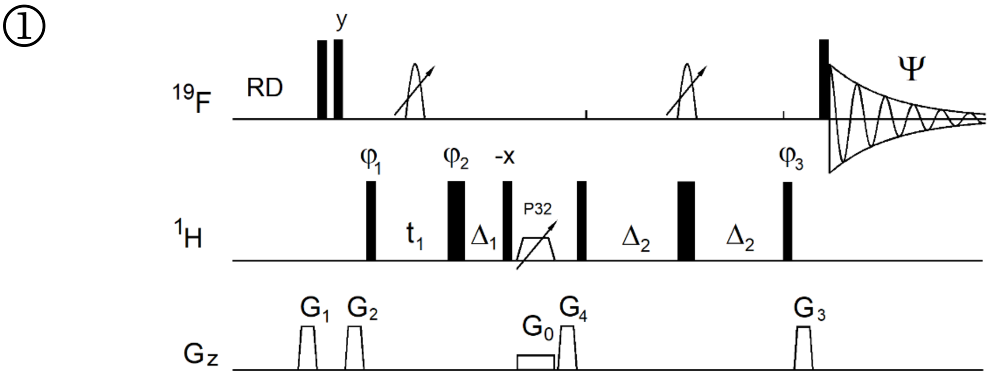

Fig. S7. Pulse sequence of the ^{19}F-detected variable-time z-filtered 2D ^{1}H, ^{19}F HETCOR experiment. The thin and thick filled rectangles represent high power 90° ^{19}F (p1) or ^{1}H (p3) and 180° (^{1}H, p4) pulses, respectively. The 1 ms adiabatic CHIRP pulses (p44) are indicated by an inclined arrow. A 20 ms 60 kHz CHIRP ^{1}H pulse (p32) was used as part of the z-filter. Unless stated otherwise, the r.f. pulses were applied from the *x*-axis. The delays were as follows: Δ_1 = p44; Δ_2 = one half of the J_{HF} evolution; $t_1(0)$, the initial t_1 evolution delay time = 0.5*in0, where in0 is the t_1 increment. G_0 = 3%; G_1 = 17%; G_2 = 31%; G_3 = 24%; G_4 = 10.0%. The following phases were used: φ_1 = x, -x; φ_2 = 4x, 4(-x); φ_3 = 2y, 2(-y); Ψ = x, 2(-x), x. States-TPPI protocol was used for sign discrimination in F_1 with the phase φ_1 incremented by 90°. Purging of ^{19}F magnetisation at the beginning of the pulse sequence by a composite 90° ^{19}F pulse and PFGs minimises the cancellation artefacts.

Polarisation transfer efficiency. The $H_i \rightarrow F$ polarisation transfer efficiency in a spin system of *n* protons coupled to proton H_i is given by the following transfer function (neglecting relaxation):

$$I_i = \sin(\pi J_{H_iF} 2\Delta_2) \prod_{j=1}^{n} \cos(\pi J_{H_iH_j} 2\Delta_2) \qquad (\mathbf{1})$$

Setting of the $2\Delta_2$ polarisation transfer interval, therefore, depends on the values of the active, J_{H_iF}, and passive, $J_{H_iH_j}$ coupling constants. In the absence of passive couplings, $2\Delta_2$ should be set to $1/(2J_{H_iF})$ yielding a transfer efficiency of 100%. For spin-systems with 1, 2 or 3 passive $J_{H_iH_j}$ coupling constants of the same size as J_{H_iF}, the optimum timing is equal to $1/(nJ_{H_iF})$, where n = 4, 5, or 6, achieving transfer efficiency of 50, 38 or 32%, respectively

In real molecules, where the J_{HF} and J_{HH} vary in size, the transfer is typically optimised for the largest J_{HF} coupling constant considering 1 or 2 passive J_{HH} passive coupling constants. In the experiments performed here, the transfer was optimised for J_{HF} = 10 Hz (active) and one J_{HH} = 10 Hz (passive) coupling constants, by setting $2\Delta_2$ = 1/(4*10) = 0.025 s (or 25 ms). Using the average J_{HF} coupling constants (in red) of aromatic protons (Table S2) and considering only the *sin* term of *Eqn* 1, the calculated transfer efficiencies (in blue) are given on the structure below.

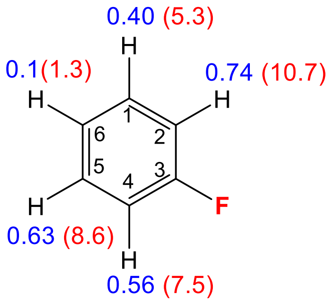

It can be seen that the transfer efficiency for individual positions reflects the sizes of J_{HF} coupling constants, nevertheless, all but the $H_6 \rightarrow F$ transfer produce satisfactory values. In these molecules only J_{HH}^{ortho} (average value 8.6 Hz) is sufficiently large to cause an additional decrease in the polarisation transfer. Such effects are clearly visible on the cross peaks for molecule **9** in the 2D ^{1}H, ^{19}F HETCOR spectrum presented in Fig. 4, where the H_2F cross peak is the most intense (no J_{HH}^{ortho}), followed by the H_5F cross peak (one J_{HH}^{ortho}), while the H_6F cross peak is too weak to be detected. Despite these effects, the sensitivity of HF correlations is not the limiting factor for the structure determination process, as commented on in the main body of the paper.

②

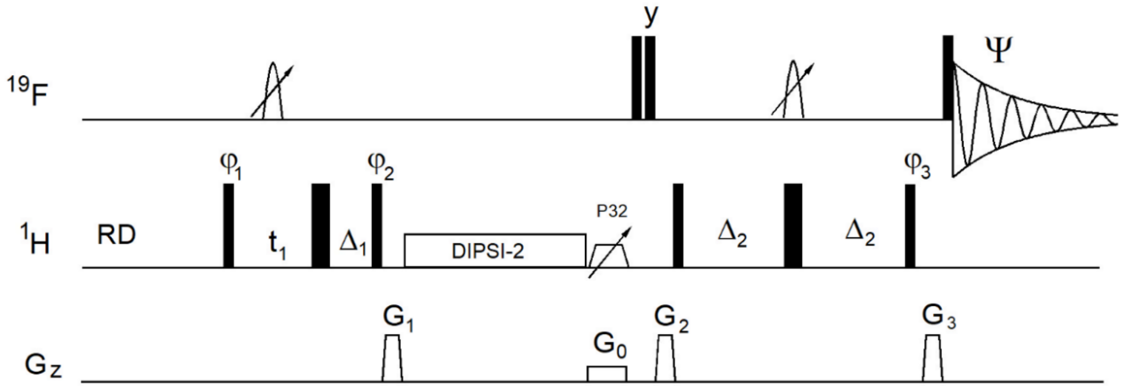

Fig. S8. Pulse sequence of ^{19}F-detected 2D ^{1}H, ^{19}F TOCSY-HETCOR experiments. The thin and thick filled rectangles represent high power 90° ^{19}F (p1) or ^{1}H (p3) and 180° (^{1}H, p4) pulses, respectively. The 1 ms adiabatic CHIRP pulses (p44) are indicated by an inclined arrow. A 20 ms 60 kHz CHIRP ^{1}H pulse (p32) was used as part of the z-filter. Unless stated otherwise, the r.f. pulses were applied from the *x*-axis. The delays were as follows: Δ_1 = p44; Δ_2 = one half of the J_{HF} evolution; $t_1(0)$ is the initial t_1 evolution delay time = 0.5*in0, where in0 is the t_1 increment. G_o = 5%; G_1 = 17%; G_2 = 31%; G_3 = 24%. The following phases were used: φ_1 = x, -x; φ_2 = 4x, 4(-x); φ_3 = 2y, 2(-y); Ψ = x, 2(-x), x, -x, 2x, -x States-TPPI protocol was used for sign discrimination in F_1 with the phase φ_1 incremented by 90°. Purging of ^{19}F magnetisation after the z-filter by a composite 90° ^{19}F pulse followed by the G_2 PFG minimises the cancellation artefacts.

Polarisation transfer efficiency. The task of evaluating the overall $H_i \rightarrow F$ polarisation transfer efficiency of the 2D ^{1}H, ^{19}F TOCSY-HETCOR experiment can be split into two parts, the $H_j \rightarrow H_i$ TOCSY transfer and the $H_i \rightarrow F$ polarisation transfer. The former depends on the nature of the proton network only, and yields efficiencies typical for 2D ^{1}H, ^{1}H TOCSY experiments; the efficiency of the latter part of the pulse sequence is given by *Eqn* **1**. The overall efficiency is the product of the efficiencies of the two parts. Because of these considerations, this experiment should be less sensitive than the 2D ^{1}H, ^{19}F HETCOR experiment. Nevertheless, depending on the proton network, an increase in the ^{19}F signal can occur. After the initial chemical shift labelling of protons, the magnetisation is spread throughout the spin system. Taking an example of a proton that has a small J_{HF} coupling constant (e.g. H_6), its magnetisation may end up on multiple protons with large J_{HF} coupling constants. Magnetisation of these protons is then in the second part of the pulse sequence transferred to fluorine, which means that the observed ^{19}F multiplet may be a superposition of several signals. This increases its intensity. Due to the antiphase nature of the HETCOR multiplets, the inner lines of these composite multiplets may be attenuated, while the most outer parts will always add up constructively, increasing their intensity. Analysing the HF cross peak of H_6 in molecule **9**, which was missing in the HETCOR experiment of Fig. S7, this proton has one large J_{H6H5} = 8.6 Hz and one small J_{H6H2} = 2.6 Hz. This makes the $H_6 \rightarrow H_5$ TOCSY transfer much more efficient than the $H_6 \rightarrow H_2$ transfer, and despite the fact that that J_{H2F} and J_{H5F} are of comparable size, the $H_6 \rightarrow H_5 \rightarrow F$ transfer pathway dominates and the H_6,F cross peak has a shape of the H_5,F cross peak. The H_6,F cross peak is clearly visible in the spectrum in Fig. 4 and the sensitivity of the 2D ^{1}H, ^{19}F TOCSY-HETCOR experiment is not the limiting factor for the structure determination process.

③

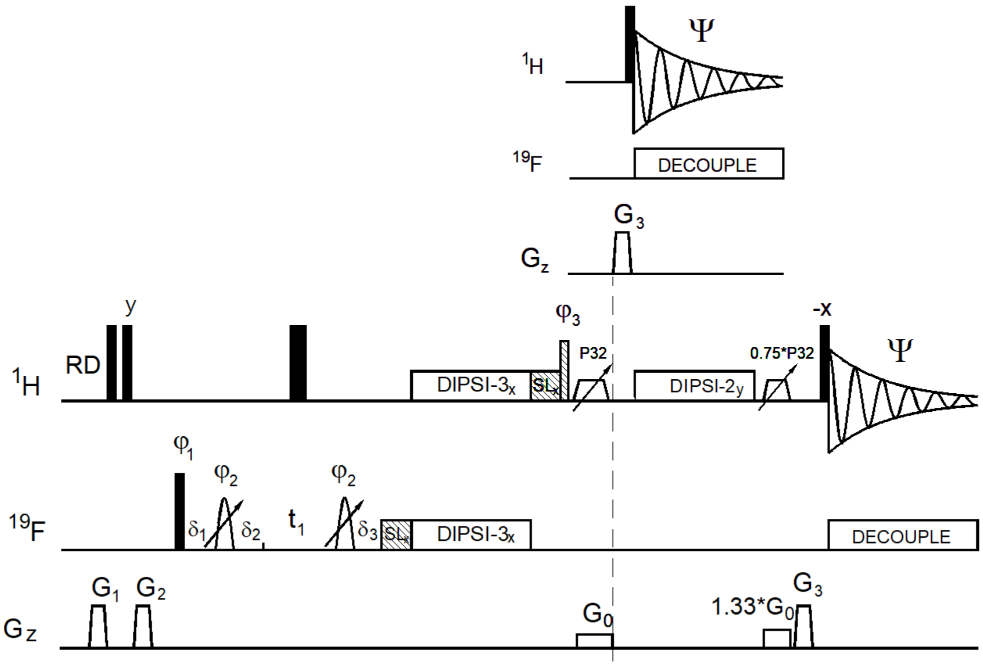

Fig. S9. Pulse sequence of ^{1}H-detected 2D ^{19}F, ^{1}H CP-DIPSI3-DIPSI2 experiments. The dashed line indicates signal acquisition before optional ^{1}H-^{1}H spin-lock. The thin and thick filled rectangles represent high power 90° (^{1}H, p1 or ^{19}F, p3) and 180° (^{1}H, p2) pulses, respectively. The 1 ms ^{19}F adiabatic CHIRP pulses (p44) are indicated by an inclined arrow. A 20 ms 60 kHz CHIRP ^{1}H pulse (p32) was used as part of the z-filter. Unless stated otherwise, the r.f. pulses were applied from the *x*-axis. The delays were as follows: δ_1 = 20μs; $\delta_2 = \delta_1 + (2/\pi)$*p3; δ_3 = p2; $t_1(0)$ is the initial t_1 evolution delay time = 0.5*in0, where in0 is the t_1 increment. G_o = 5%; G_1 = 17%; G_2 = 31%; G_2 = 66%. The following phases were used: φ_1 = y, -y; φ_2 = 4x, 4(-x); φ_3 = 2y, 2(-y); Ψ = x, 2(-x), x. States-TPPI protocol was used for sign discrimination in F_1 with the phase φ_1 incremented by 90°. Purging of ^{19}F magnetisation at the beginning of the pulse sequence by a composite 90° ^{19}F pulse and PFGs minimises the cancellation artefacts.

Polarisation transfer efficiency. This experiment contains two spin-lock periods, the first mediates the heteronuclear (H → F), while the second mediates the homonuclear (H → H) transfer. In an isolated X, Y spin system, the efficiency of in-phase magnetisation transfer between the two spin-locked spins is proportional to $sin(0.5\pi J_{xy}\tau)$; the maximum (100%) therefore occurs for $\tau = 1/J_{XY}$. It is therefore advisable to set the polarisation transfer interval for the first transfer close to $\tau = 1/J_{HF}^{max}$, where J_{HF}^{max} is the largest HF coupling constant. Note that in this experiment, the H → H transfer is already taking place during the H → F spin-lock, and therefore the protons with small or zero J_{HF} coupling constants may appear in the spectra even without the additional pure H → H transfer. This latter transfer period reinforces signal intensities and its length and efficiency depends on the nature of the proton spin system. A typical value of around 50 ms is recommended.

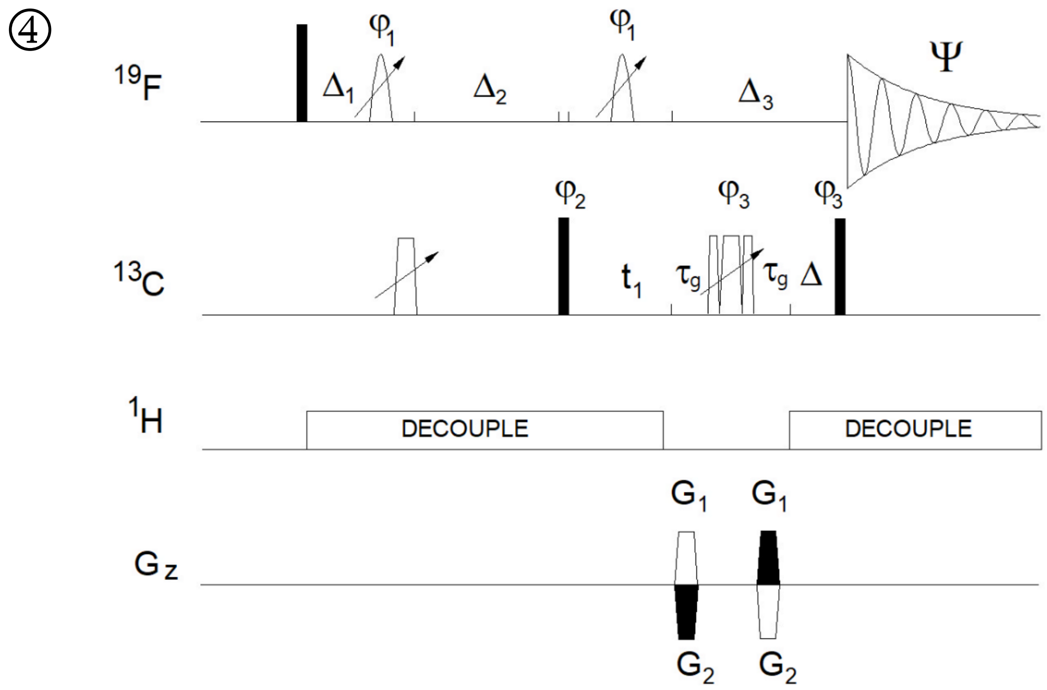

Fig. S10. Pulse sequence of a ^{19}F-detected 2D ^{19}F, ^{13}C (^{15}N) HMBC experiment optimised for $^{n}J_{FC}$ ($^{n}J_{FN}$) correlations. The thin filled rectangles represent high power 90° ^{19}F (p1) or ^{13}C (^{15}N) (p3) pulses. The 1 ms adiabatic CHIRP pulses (p44) applied to ^{19}F are indicated by an inclined arrow. A 500 μs CHIRP pulse (p14) and 2 ms composite CHIRP pulse (p24) were applied to ^{13}C (^{15}N). Unless stated otherwise, the r.f. pulses were applied from the *x*-axis. The delays were as follows: d6 = 0.25/$^{n}J_{FX}$; Δ = p44; Δ_3 =2*p16+2*d16+p24+Δ+8μs; Δ_1 = d6 – Δ_3/2; Δ_2 = d6 + Δ_3/2 – p14 + (2/π)*p1; t_1(0) is the initial t_1 evolution delay time = 0.5*in0, where in0 is the t_1 increment. G_1 = 80%; G_2 = cnst30*G_1, where cnst30 = (1-sfo2/sfo1)/(1+sfo2/sfo1) and sfo1 and sfo2 are ^{19}F and ^{13}C (^{15}N) frequencies, respectively. φ_1 = 2x, 2(-x); φ_2 = x, -x; φ_3 = 4x, 4(-x); Ψ = 2(x, -x), 2(-x, x). Echo-anti echo protocol was used with PFGs changing sign between real and imaginary increments. Phases φ_2 and Ψ were incremented by 180° together with the PFG sign change.

Polarisation transfer efficiency. The signal intensity in this experiment is proportional to $sin(\pi J_{FC}(\Delta_1 + \Delta_2)$. For spin systems with one ^{19}F atom, there are no passive coupling constants that could decrease the efficiency of the polarisation transfer. At the same time, ^{1}H decoupling ensures that proton-fluorine couplings do not interfere either. The HMBC experiment can therefore achieve high levels of transfer efficiency by setting the evolution interval to $\Delta_1 + \Delta_2 = 1/(2J_{FC}^{max})$. In experiments performed here, the transfer was optimised for a 20 Hz J_{FC} coupling constant, yielding $\Delta_1 + \Delta_2 = 25\ ms$. Using average $^{n}J_{FC}$ coupling constants (in red) for the aromatic carbons (Table S3), the transfer efficiencies for a 25 ms evolution interval are stated (in blue) on the structure below.

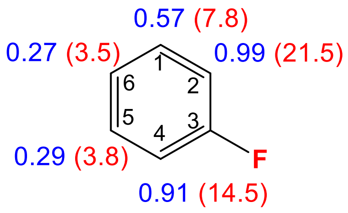

It can be seen that transfer efficiency at individual positions reflects the sizes of J_{FC} coupling constants, nevertheless, even a ~6 fold smaller J_{FC6} (3.5 Hz) produced transfer only 3.7 times lower than the J_{FC2} of 21.53 Hz. The trends in cross peak intensities outlined here are clearly visible in the 2D ^{19}F, ^{13}C HMBC spectrum of molecule **9** in Fig. 4. To rebalance the intensities in favour of cross peaks for carbons with smaller J_{FC} constants, the evolution interval could be lengthened, e.g. set to $\Delta_1 + \Delta_2 = 1/(1.4J_{FC}^{max})$ without running the risk of zeroing accidently cross peaks mediated by large J_{FC} coupling constants. Overall, due to the absence of passive coupling constants, this crucial experiment for the structure determination process performs well despite the natural spread of $^{n}J_{FC}$ coupling constants.

④'

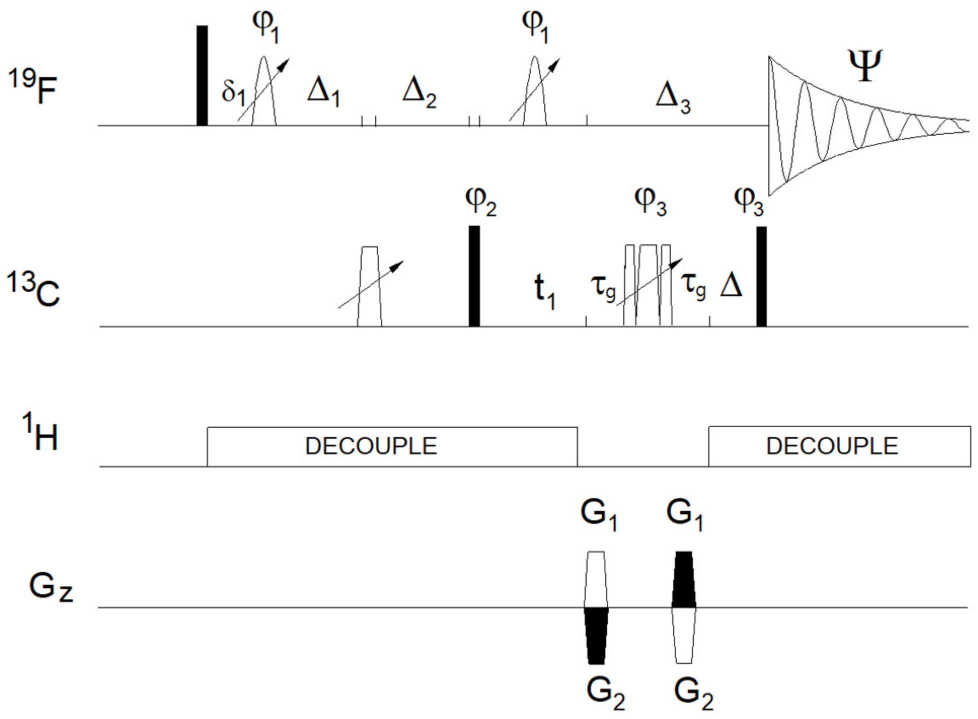

Fig. S11. Pulse sequence of ^{19}F-detected 2D ^{19}F, ^{13}C HMBC experiment optimised for $^{1}J_{FC}$ correlations. The thin filled rectangles represent high power 90° ^{19}F (p1) or ^{13}C (p3) pulses. The 1 ms adiabatic CHIRP pulses (p44) applied to ^{19}F are indicated by an inclined arrow. A 500 μs CHIRP pulse (p14) and 2 ms composite CHIRP pulse (p24) were applied to ^{13}C. Unless stated otherwise, the r.f. pulses were applied from the *x*-axis. The delays were as follows: d6 = 0.5/$^{1}J_{FC}$; Δ = p44; δ_1 = 20μs; Δ_3 =2*p16+2*d16+p24+Δ+8μs; Δ_1 = (Δ_3 – p14 – d6)/2 + (2/π)*p1 + δ_1; Δ_2 = (Δ_3 – p14 + d6)/2; $t_1(0)$ is the initial t_1 evolution delay time = 0.5*in0, where in0 is the t_1 increment. G_1 = 80%; G_2 = cnst30*G_1, where cnst30 = (1-sfo2/sfo1)/(1+sfo2/sfo1) and sfo1 and sfo2 are ^{19}F and ^{13}C frequencies, respectively. φ_1 = 2x, 2(-x); φ_2 = x, -x; φ_3 = 4x, 4(-x); Ψ = 2(x, -x), 2(-x, x). Echo-anti echo protocol was used with PFGs changing the sign between real and imaginary increments. Phases φ_2 and Ψ were incremented by 180° together with the sign change.

Polarisation transfer efficiency. The signal intensity in this experiment is proportional to $sin(\pi ^1J_{FC}d_6)$. Depending on the spread of $^{1}J_{FC}$ values, this experiment can be optimised very well, with $d6 = 1/(2\ ^1J_{FC}^{max})$. For the average $^{1}J_{FC}$ for these compounds (245.94 Hz, Table S2), d6= 2.03 ms, yielding 100% transfer efficiency.

⑤

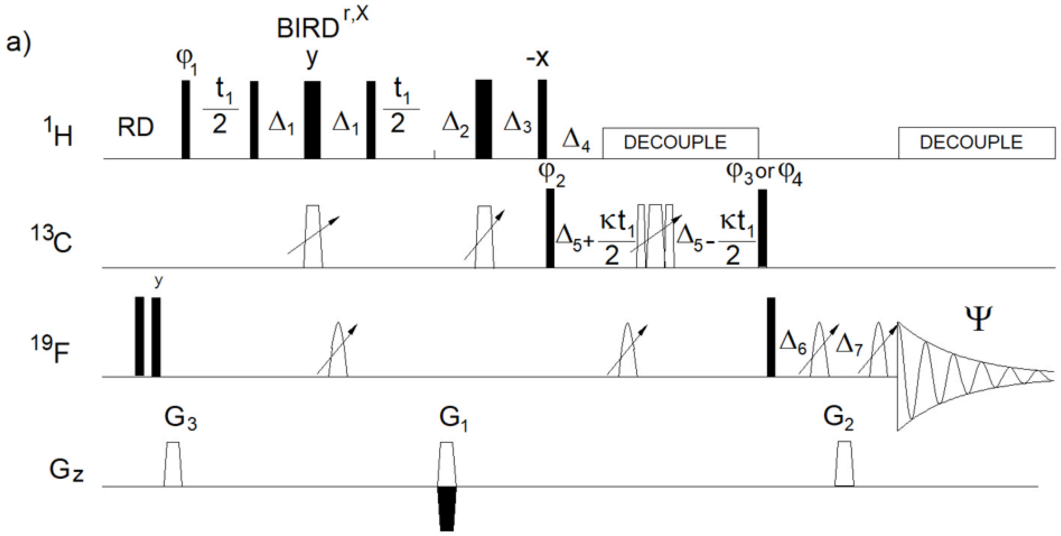

Fig. S12. Pulse sequence of [1]H-detected 2D H[1]C[n]F experiment for the correlation via $^{1}J_{HC}$ and $^{n}J_{FC}$. The thin and thick filled rectangles represent high power 90° (^{1}H, p1 or ^{19}F, p3) and 180° (^{1}H, p2) pulses, respectively. The 1 ms adiabatic CHIRP pulses (p44) applied to ^{19}F are indicated by an inclined arrow. A 500 µs CHIRP pulse (p14) and 2 ms composite CHIRP pulse (p24) were applied to [13]C. Unless stated otherwise, the r.f. pulses were applied from the *x*-axis. The delays were as follows: d2 = 0.25/$^{1}J_{HC}$; d3 = 0.5/$^{1}J_{HC}$; d4 = 0.25/$^{n}J_{FC}$; d6 = cnst1/$^{1}J_{HC}$, where cnst1=0.5 for CH and 0.25 for CH_2 groups; Δ_1 = d3 – p14/2; Δ_2 = d2 – p14/2-p16 - d16; Δ_3 = d2 – p14/2 - $2t_1(0)$; Δ_4 = d6; Δ_5 = d4; Δ_6 = p16 + d16 – (2/π)p1 + 4µs; Δ_7 = p16 + d16 + 4µs, where p16 and d16 are the PFG length and the recovery time, respectively. G_1 = 40 %; G_2 = 42.51 %; G_3 = 13%; φ_1 = y, -y; φ_2 = 4x,4(-x); φ_3 = 2x, 2(-x); φ_4 = 2y, 2(-y); Ψ = x, 2(-x), x, -x, 2x, -x. Echo-anti echo protocol was used with G_1 changing sign between real and imaginary increments. Phases φ_1 and Ψ were incremented by 180° together with the sign change. Two interleaved experiments are acquired applying either φ_3 or φ_4 phase to the last 90° [13]C pulse.

Polarisation transfer efficiency. The H → C→ F polarisation transfer pathway of this pulse sequence (neglecting relaxation) is given by the following transfer function:

A | **B** | **C** | **D**

$$I_i = \sin(\pi\, ^{1}J_{CH} d_3) \times \sin(\pi\, ^{1}J_{CH} d_6) \prod_{j=0}^{n} \cos^{j}(\pi\, ^{1}J_{CH} d_6) \times sin(\pi J_{FC} 2 d_4), \quad (\mathbf{2})$$

where *j* = 0,1 or 2 for CH, CH_2 or CH_3 carbons, respectively. This equation contains four terms, A, to D. The first, **A**, describes the H → C polarisation transfer via $^{1}J_{CH}$ coupling constants and can be optimised to achieve near 100% transfer efficiency by setting d_3 to 1/(2$^{1}J_{CH}$). The terms **B** and **C** describe refocusing of the antiphase ^{13}C magnetisation with respect to the $^{1}J_{CH}$ coupling constants. For CH moieties these can be optimised to near 100% transfer efficiency by setting d_6 to 1/(2$^{1}J_{CH}$). For CH_2 and CH_3 carbons, a transfer efficiency of 50 and 38% is achieved by setting d_6 to 1/(4$^{1}J_{CH2}$) and 1/(5$^{1}J_{CH3}$), respectively. For molecules with one ^{19}F atom, a 100% efficiency is achieved for the term **D** by setting d_4 to $1/(4J_{FC}^{max})$. To enhance the intensity of cross peaks with smaller J_{FC} couplings, the Δ_5 interval can be set to $1/(2.8J_{FC}^{max})$ Alternatively, because this experiment is typically acquired after the HMBC experiment, which provides exact values of $^{n}J_{FC}$ couplings, a bespoke optimisation can be performed. The spectrum in Fig. 4 was acquired with d_4 equal to 50 ms, which using the average coupling constants for carbons C2, C5 and C6 (Table S3), yielded the transfer efficiencies of 46, 93 and 89 % for the **D** term, respectively. These predictions agree with the intensity of cross peaks of compound **9** in Fig. 4d, where the FH_2 cross peak has approximately half the intensity of the FH_5 or FH_6 cross peaks. Although less sensitive than the HMBC experiments, sufficient signal was obtained for the compounds above the sensitivity threshold.

Table S1. Parameters of the NMR experiments performed on the reaction mixture[a].

Parameter /experiment	RD | NS /s | -	J evolution delay /ms	SW_1 | SW_2 /ppm	TD_1 | TD_2 /points	AQ_1 | AQ_2 /ms | /s	Overall time /h
1D ^{19}F	4 | 2k	-	147.6	256k	1.89	3.3
^{19}F-detected VT, z-filtered $^{1}H,^{19}F$ HETCOR	1.6 | 24	25	8.0 | 62.3	320 | 32k	25 | 0.35	4.19
^{19}F-detected $^{1}H,^{19}F$ TOCSY-HETCOR	1.6 | 24	60 (H→H) 25	8.0 | 62.3	320 | 32k	25 | 0.35	4.19
2D ^{19}F, ^{1}H CP-DIPSI3-DIPSI2	2 | 16	90 (F→H) 60 (H→H)	16 | 12	768 | 8k	51 | 0.68	9.2
2D $^{19}F,^{13}C$ HMBC ($^{n}J_{FC}$)	2 | 16	25	120 | 99.6	768 | 32k	25 | 0.35	9.2
2D $^{19}F,^{15}N$ HMBC ($^{n}J_{FN}$)	2 | 8	167	34.6 | 200	1k | 32k	5.1 | 0.84	4.9
(3, 2)D $H^{1}C^{n}F$	1.6 | 48	100	8.0 | 34.6	2 × 256 | 32k	26.7 | 0.84	11.6
2D ^{1}H, ^{15}N HSQC[b]	2 | 16	5.6	100 | 20.2	512 | 1k	31.6 | 0.127	5
^{19}F DOSY[c,d]	2 | 256	-	- | 34.6	16 | 128k	| 3.4	4

[a] Acquired at 500 MHz; [b] Acquired at 800 MHz; [c] For a wider range of ^{19}F resonances the use of adiabatic pulses is recommended[1, 2] [d] Acquired using a BRUKER program, ledbpgp2s, modified according to ref.[3] The diffusion time was set to 100ms and bipolar de-/rephasing gradients (1 ms) were applied at the strength of 5 to 95 % of the nominal value of 56 Gauss/cm increasing linearly

Table S2. Summary of $^{n}J_{HF}$ coupling constants (/Hz) for the 13 characterised aromatic compounds.

Proton<br>Compound	**H1**<br>$^{4}J_{HF}$	**H2**<br>$^{3}J_{HF}$	**H4**<br>$^{3}J_{HF}$	**H5**<br>$^{4}J_{HF}$	**H6**<br>$^{5}J_{HF}$
**1***	-	11.5	-	8.9	0.8
**2**	-	10.9	-	-	1.5
**3**	-	10.2	-	-	1.8
**4***	4.4	8.5	8.5	4.4	-
**5**	6.1	10.9	-	-	0.9
**6***	-	8.5	6.5	6.5	-
**7***	-	10.5	-	7.3	< 0.5
**8***	-	10.8	-	8.7	1
**9**	-	10.8	-	9.3	1.2
**10**	-	10.9	-	-	1.5
**11**	-	10.5	-	8	0.5
**12**	-	10.6	-	-	1.7
**13**	-	10.4	-	-	1.8
Average	5.25	10.71	7.50	8.64	1.27
Std dev	±0.85	±0.34	±1.00	±0.88	±0.43

* values given are the first order approximations as protons are strongly coupled for these compounds; highlighted coupling constants of compounds 4-6 were excluded from calculating the average values because their structures differ significantly from the rest of the compounds.

Table S3. Summary of $^{1,n}J_{FC}$ coupling constants (/Hz) of 13 characterised aromatic compounds.

Carbon	**C1**	**C2**	**C3**	**C4**	**C5**	**C6**	**C7 (COOH)**
Compound	$^{3}J_{FC}$	$^{2}J_{FC}$	$^{1}J_{FC}$	$^{2}J_{FC}$	$^{3}J_{FC}$	$^{4}J_{FC}$	$^{4}J_{FC}$
**1**	6.1	20.4	241.4	12.9	2.5	3.2	2.9
**2**	7.2	20.7	242.5	16.1	4.3	3.2	3.2
**3**	10.7	22.9	245.0	16.1	5.5	3.2	
**4**	7.9	23.2	234.6	23.2	7.9	2.5	
**5**	12.2	23.2	259.3	13.2	3.6	3.2	3.2*
**6**	10.7	26.1	238.9	22.9	8.6	2.5	-
**7**	6.4	19.7	251.1	12.9	N.D.	3.9	3.2
**8**	7.5	23.2	245.3	12.9	3.6	3.2	
**9**	8.6	22.2	243.9	12.9	3.6	3.6	
**10**	6.8	20.7	242.8	16.5	3.2	3.2	2.9
**11**	8.9	21.1	253.6	12.9	N.D.	3.9	
**12**	6.8	20.0	247.8	16.1	3.2	3.9	3.6
**13**	8.9	24.3	246.0	16.1	4.3	3.4	
Average	7.79	21.53	245.94	14.52	3.77	3.49	3.15
Std dev	±1.38	±1.46	±3.68	±1.65	±0.85	±0.31	0.27

* this is a $^{3}J_{FC}$;highlighted coupling constants of compounds 4-6 were excluded from calculating the average values because their structures differ significantly from the rest of the compounds.

Table S4. Summary of ^{13}C-induced ^{19}F isotopic shifts[a] (/ppb) for the 13 characterised aromatic compounds.

Compound	**C1**	**C2**	**C3**	**C4**	**C5**	**C6**	**C7**
**1**	5.8	24.0	78.7	25.9	4.2	2.7	0.8
**2**	5.1	24.1	80.3	25.2	5.1	3.9	0.0
**3**	4.1	22.8	81.6	25.4	4.5	4.1	0.0
**4**	6.6	26.0	83.3	26.0	6.6	7.7	0.0
**5**	4.7	27.1	87.1	21.0	4.7	3.5	0.0
**6**	5.7	24.3	84.7	25.4	6.4	7.6	0.0
**7**	5.1	23.7	85.7	26.4	N.D.	3.2	0.9
**8**	5.1	24.1	80.3	25.2	5.1	3.9	0.9
**9**	5.2	23.4	80.4	26.5	4.4	3.7	0.0
**10**	6.3	25.6	82.3	26.4	5.5	4.7	1.3
**11**	4.5	22.7	86.6	26.2	N.D.	3.0	0.0
**12**	6.4	27.3	83.2	24.7	4.1	6.4	2.2
**13**	6.6	24.5	83.0	26.4	5.5	5.0	0.0
Average	5.5	24.6	82.9	25.4	5.1	4.6	0.5
Stdev	±0.8	±1.4	±2.5	±1.4	±0.8	±1.6	0.7

[a] calculated as $10^3 \times [\nu_\delta(^{19}F_{12C}) - \nu_\delta(^{19}F_{13C})]/ \nu_L(^{19}F)$ where ν_δ and ν_L are given in Hz and MHz respectively; highlighted coupling constants of compounds 4-6 were excluded from calculating the average values because their structure differs significantly from the rest of the compounds.

Table S5. Summary of ^{19}F, ^{1}H, ^{13}C and ^{15}N NMR parameters for the 13 characterised aromatic compounds.

Compound	$^{1}H/^{19}F$ NMR parameters[a-d]	$^{13}C/^{19}F$ NMR parameters[c,e,f,g]
**1**	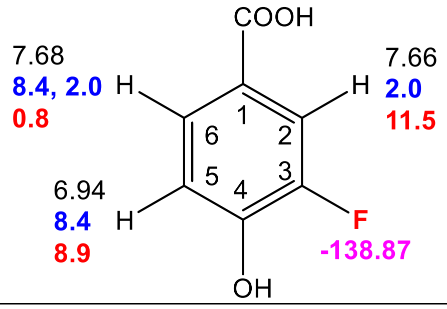	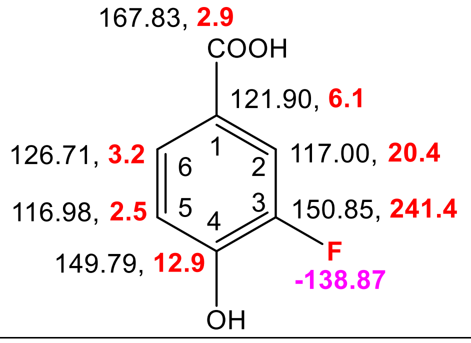
**2**	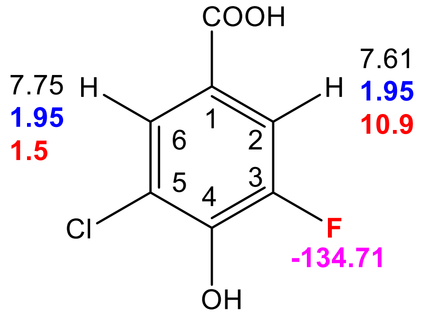	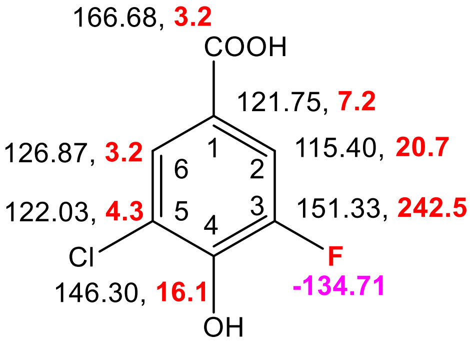
**3**	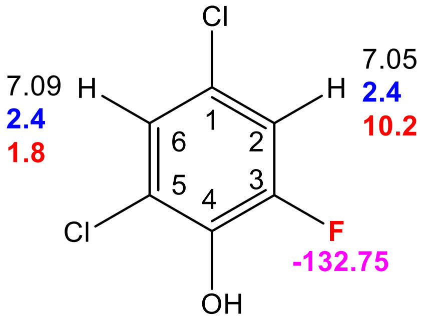	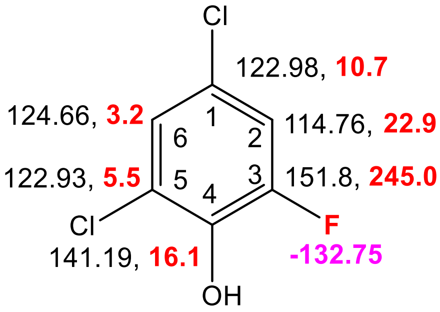
**4**[h]	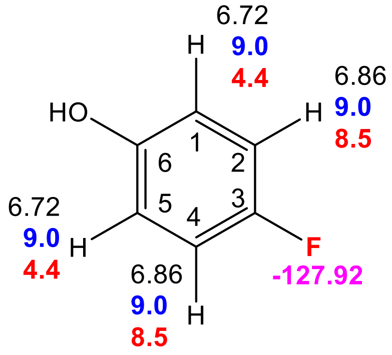	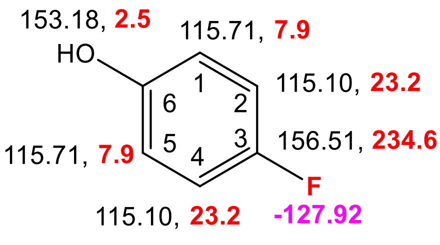

<table>
<tr>
<td>5[i]</td>
<td>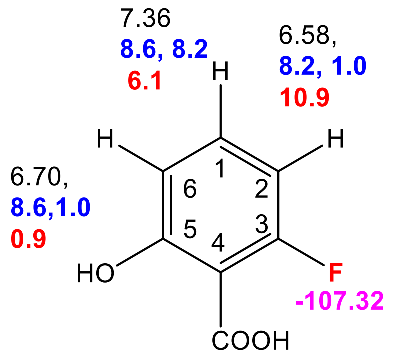
</td>
<td>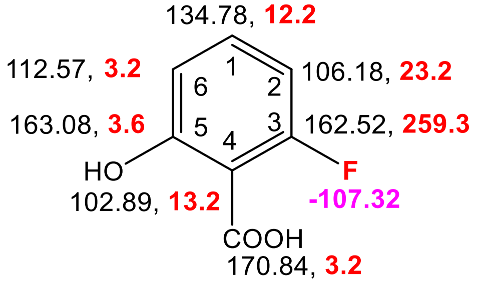
</td>
</tr>
<tr>
<td>6[j]</td>
<td>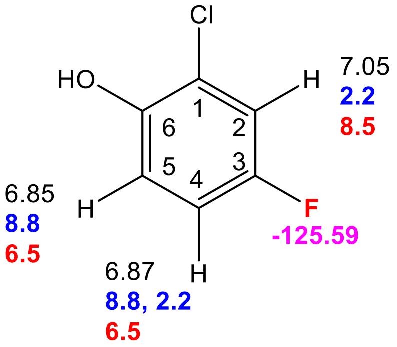
</td>
<td>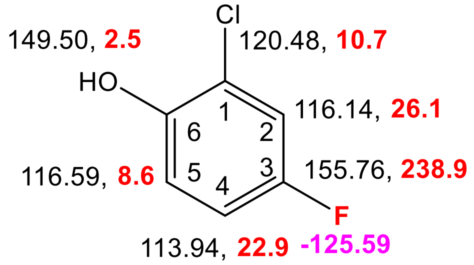
</td>
</tr>
<tr>
<td>7[k]</td>
<td>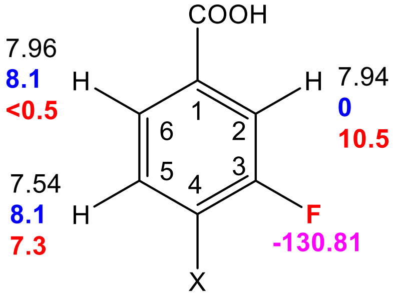
</td>
<td>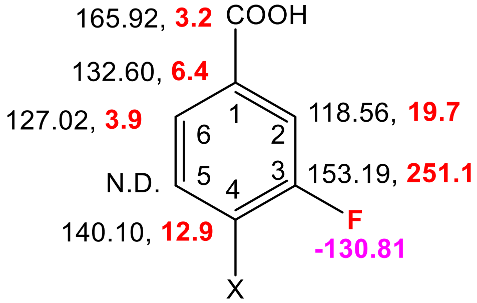
</td>
</tr>
<tr>
<td>8[l]</td>
<td>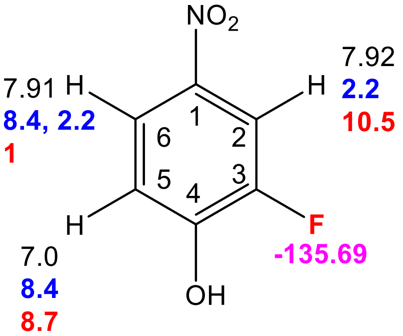
</td>
<td>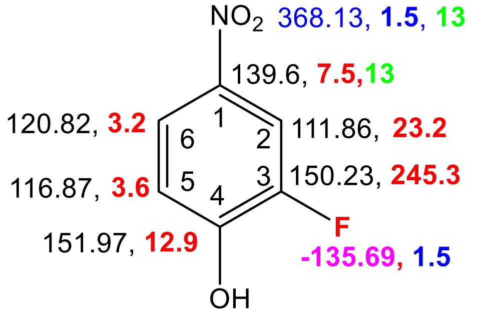
</td>
</tr>
<tr>
<td>9</td>
<td>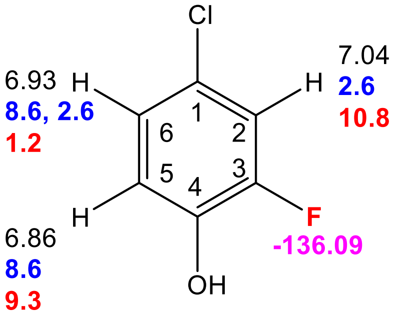
</td>
<td>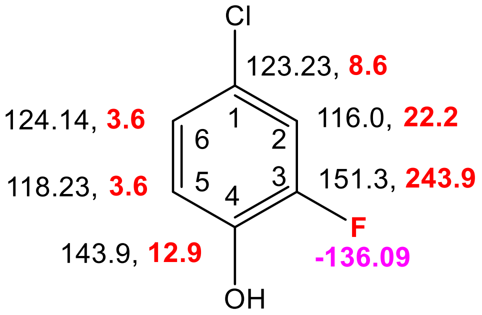
</td>
</tr>
</table>

**10**	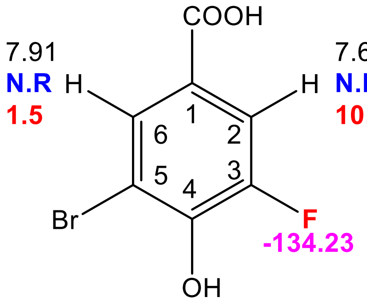	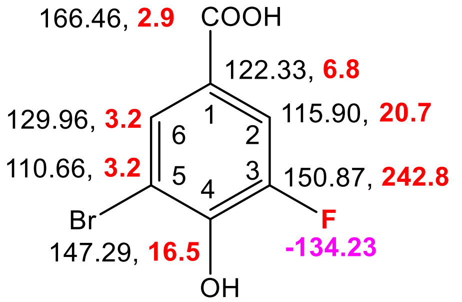
**11**	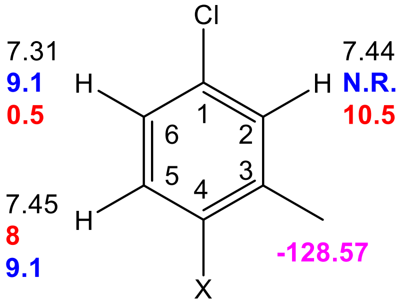	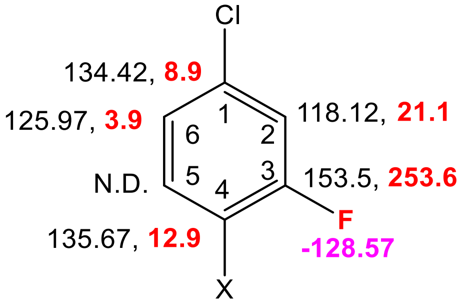
**12**[m]	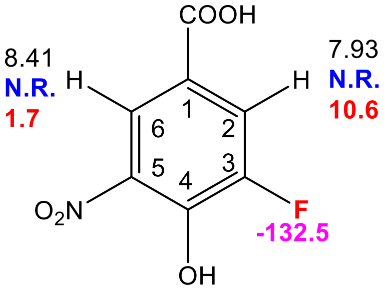	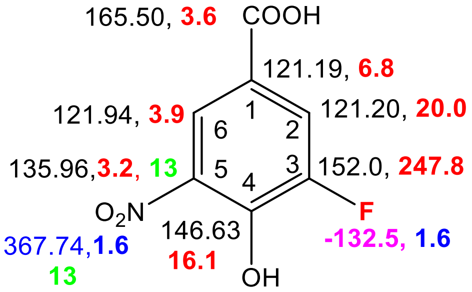
**13**[n]	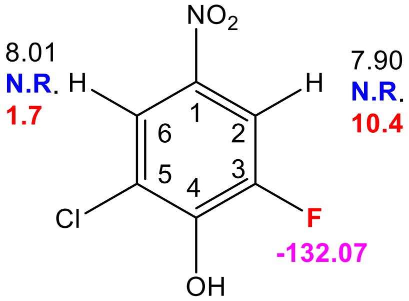	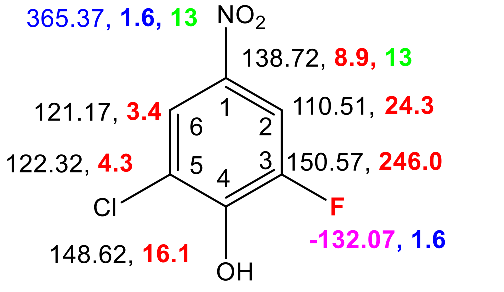

[a] δ_H/ppm (black); [b] J_{HH}/Hz (blue); [c] δ_F/ppm (magenta); [d] J_{HF}/Hz (red); [e] δ_F/ppm (black); [f] J_{FC}/Hz (red); [g] J_{FN}/Hz and δ^{15}N/ppm (cyan); [l,m,n] J_{NC}/Hz (green); [h,i,l] literature chemical shift data for compounds: **4**,[4] **5**,[5] **6** (https://sdbs.db.aist.go.jp/sdbs/cgi-bin/landingpage?sdbsno=4176), **8**;[6] [j] J_{HF} and J_{HH} were determined as the first order approximation of a strongly coupled spin system. [k] X stands for unknown; N.D. – not detected. N.R. – not resolved

References

1. M. Foroozandeh, M. Nilsson and G. A. Morris, *Journal of Magnetic Resonance*, 2019, **302**, 28-33.
2. N. Khaneja, *Journal of Magnetic Resonance*, 2017, **282**, 32-36.
3. M. D. Pelta, G. A. Morris, M. J. Stchedroff and S. J. Hammond, *Magnetic Resonance in Chemistry*, 2002, **40**, S147-S152.
4. M. J. Zhang, H. X. Li, H. Y. Li and J. P. Lang, *Dalton Transactions*, 2016, **45**, 17759-17769.
5. J. M. Silla, C. J. Duarte, R. Rittner and M. P. Freitas, *Rsc Advances*, 2013, **3**, 25765-25768.
6. R. D. Chambers, J. Hutchinson, M. E. Sparrowhawk, G. Sandford, J. S. Moilliet and J. Thomson, *Journal of Fluorine Chemistry*, 2000, **102**, 169-173.
